# Borylation of fluorinated arenes using the boron-centred nucleophile B(CN)_3_^2–^ – a unique entry to aryltricyanoborates†
†Electronic supplementary information (ESI) available: Additional tables, experimental information, analytical data and details of the DFT calculations. CCDC 1548893–1548910. For ESI and crystallographic data in CIF or other electronic format see DOI: 10.1039/c7sc02249b



**DOI:** 10.1039/c7sc02249b

**Published:** 2017-06-26

**Authors:** Johannes Landmann, Philipp T. Hennig, Nikolai V. Ignat’ev, Maik Finze

**Affiliations:** a Institut für Anorganische Chemie , Institut für nachhaltige Chemie & Katalyse mit Bor (ICB) , Julius-Maximilians Universität Würzburg , Am Hubland , 97074 Würzburg , Germany . https://go.uniwue.de/finze-group ; Email: maik.finze@uni-wuerzburg.de; b Merck KGaA , Frankfurter Strasse 250 , 64293 Darmstadt , Germany

## Abstract

The potassium salt of the boron-centred nucleophile B(CN)_3_^2–^ (**1**) readily reacts with perfluorinated arenes, such as hexafluorobenzene, decafluorobiphenyl, octafluoronaphthalene and pentafluoropyridine, which results in KF and the K^+^ salts of the respective borate anions with one {B(CN)_3_} unit bonded to the (hetero)arene. An excess of K_2_ **1** leads to the successive reaction of two or, in the case of perfluoropyridine, even three C–F moieties and the formation of di- and trianions, respectively. Moreover, all of the 11 partially fluorinated benzene derivatives, C_6_F_6–*n*_H_*n*_ (*n* = 1–5), generally react with K_2_ **1** to give new tricyano(phenyl)borate anions with high chemo- and regioselectivity. A decreasing number of fluorine substituents on benzene results in a decrease in the reaction rate. In the cases of partially fluorinated benzenes, the addition of LiCl is advantageous or even necessary to facilitate the reaction. Also, pentafluorobenzenes R–C_6_F_5_ (R = –CN, –OMe, –Me, or –CF_3_) react *via* C–F/C–B exchange that mostly occurs in the *para* position and to a lesser extent in the *meta* or *ortho* positions. Most of the reactions proceed *via* an S_N_Ar mechanism. The reaction of 1,4-F_2_C_6_H_4_ with K_2_ **1** shows that an aryne mechanism has to be considered in some cases as well. In summary, a wealth of new stable tricyano(aryl)borates have been synthesised and fully characterized using multi-NMR spectroscopy and most of them were characterised using single-crystal X-ray diffraction.

## Abstract 2

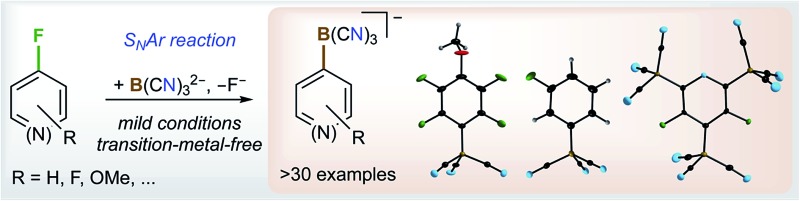
S_N_Ar reactions of the tricyanoboryl dianion B(CN)_3_^2–^ with selected fluoroarenes and perfluoropyridine resulted in a wealth of new tricyanoarylborate anions in high yields with high chemo- and regioselectivity.

## Introduction

**Scheme 1:**
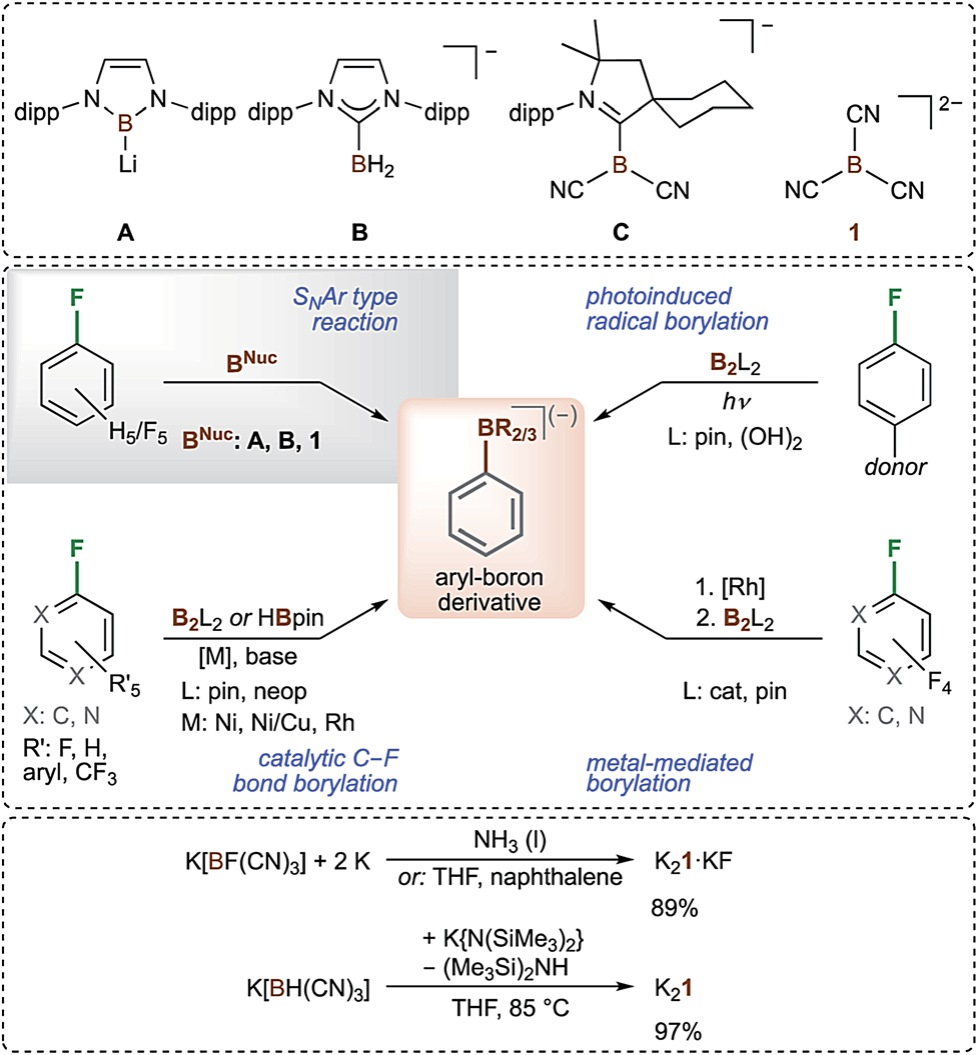
Selected boron-centred nucleophiles (top), the different types of conversion of C–F into C–B moieties of arenes and heteroarenes (middle), and convenient syntheses of K_2_ **1** (bottom); (dipp = 2,6-diisopropylphenyl, cat = catecholato, pin = pinacolato, and neop = neopentyl glycolato).

Anionic boron-centred nucleophiles are of growing interest as boron centres are usually electrophilic (Lewis-acidic). Therefore, boron-centred nucleophiles have unusual reactivity and are expected to have large synthetic potential.^1–4^ The isolation of the first boron-centred nucleophile in 2006, the lithiated boryl anion **A** that is stable in THF at –45 °C for months, was an important development in this field that stimulated intensive further research (Scheme 1).^5^ The chemistry of the lithiated boryl anion **A** and closely related species^3,6–8^ was studied in detail, and these compounds were found to be versatile starting compounds for the introduction of boryl moieties.^1,3–14^ Although some other boron-centred nucleophiles have been described, there is still a limited number of them.^15–26^ Boryl anions were found to be stabilized by carbene ligands such as NHCs (NHC = N-heterocyclic carbene) and cAACs (cAAC = cyclic (alkyl)(amino)carbene), as demonstrated in **B** ^27^ and **C** ^28^ (Scheme 1). An alternative approach for the stabilization of boron-centred nucleophiles is the introduction of cyano groups at the boron atom, as demonstrated in **C** ^28^ and the tricyanoborate dianion B(CN)_3_^2–^ (**1**).^29–32^ The latter is the only dianionic boryl anion known to date, and its alkali metal salts are indefinitely stable at room temperature under an inert atmosphere. The first syntheses towards the alkali metal salts of **1** started from M[B(CN)_4_] (M = alkali metal).^29^ Recently, we reported convenient, high-yield and large-scale entries towards the salts of **1** starting from the readily available tricyanoborates M[BF(CN)_3_]^33,34^ and M[BH(CN)_3_]^35,36^ (Scheme 1).^30–32^ The unprecedented formation of dianion **1** from [BH(CN)_3_]^–^ is the first example of the deprotonation of a hydridoborate anion.^31,32^ The conversion of a hydridoborane into monoanion **C** is the only related reaction^28^ known to date.

As mentioned previously, boron-centred nucleophiles have large synthetic potential. Surprisingly, only very few examples of reactions of such nucleophiles with fluorinated arenes have been reported. Boryl lithium **A** was found to react with C_6_F_6_ or C_6_H_5_F to obtain the corresponding monoborylated benzenes.^6^ Similar reactions have been reported for **B** ^27^ and **1** ^30^ with C_6_F_6_ only (Scheme 1). In general, only a limited number of transformations of C–F into C–B bonds of arenes or heteroarenes are known. They are either metal-catalyzed^37–42^ or metal-mediated^43^ reactions, or photoinduced radical borylations^44,45^ (Scheme 1).

Herein, we report on the S_N_Ar reactions of the boryl dianion B(CN)_3_^2–^ (**1**) with selected fully and partially fluorinated arenes^46–51^ including perfluorinated pyridine, naphthalene and biphenyl as well as fluorinated arenes with a functional group. All of the reactions proceeded *via* the exchange of fluorine with the tricyanoboryl moiety and most of them were chemo- and regioselective. Even multiple exchange reactions that provide access to bis- and tris(tricyanoborate) anions have been achieved.

## Results and discussion

### Reactions of K_2_B(CN)_3_ (K_2_ **1**) with perfluoropyridine

**Fig. 1:**
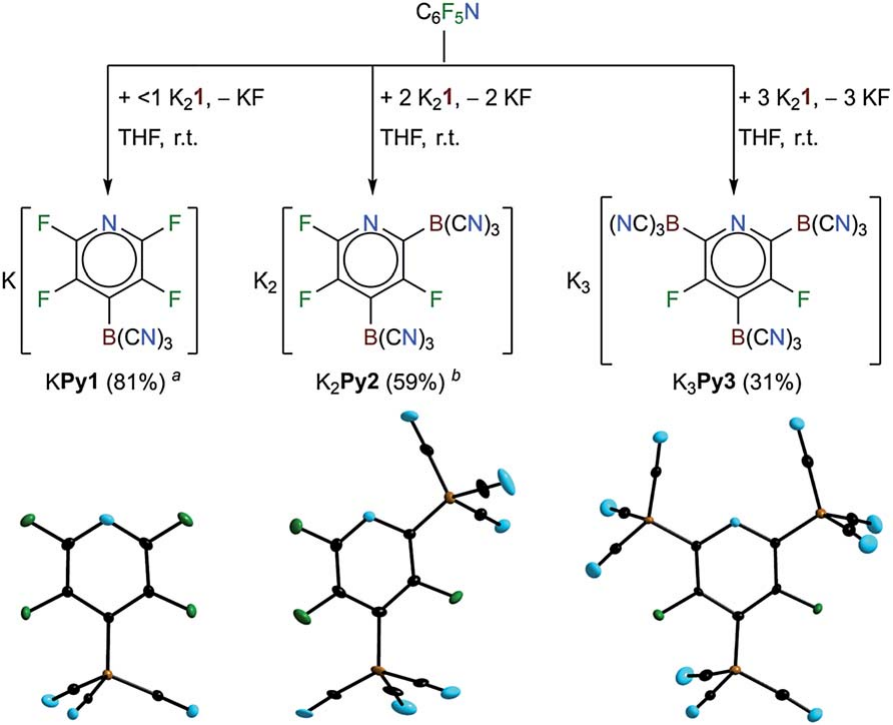
Top: the reactions of K_2_ **1** with pentafluoropyridine to give **Py1**, **Py2** and **Py3** (^*a*^contained 15% other tricyano(fluoropyridinyl)borates; ^*b*^contained 15% K_3_ **Py3** and 10% another tricyano(fluoropyridinyl)borate). Bottom: the anions in crystals of their K^+^ salts (the displacement ellipsoids are at the 25% probability level, except for **Py3** where they are at 50%).

As outlined in the introduction, K_2_B(CN)_3_ (K_2_ **1**) was found to react with an excess of C_6_F_6_ at room temperature in THF to give the salts of the [C_6_F_5_B(CN)_3_]^–^ anion (**B6a**) in 67% yield.^30^ A related reaction of perfluoropyridine with K_2_ **1** was found to start at temperatures below 0 °C, as evidenced by the rapid decolorization of the suspension. The [4-{(NC)_3_B}-C_5_F_4_N]^–^ anion (**Py1**) was obtained as the main product of the reaction with an excess of perfluoropyridine (Table 1 and Fig. 1). The exchange of the fluorine in the 4-position is typical for the S_N_Ar reactions of C_5_F_5_N.^52–55^ The dianion [2,4-{(NC)_3_B}_2_-C_5_F_3_N]^2–^ (**Py2**), a different isomer of **Py1**, most likely [2-{(NC)_3_B}-C_5_F_4_N]^–^, and small amounts (<1%) of further tricyano(fluoropyridinyl)borate anions, including the trianion [2,4,6-{(NC)_3_B}_3_-C_5_F_3_N]^3–^ (**Py3**), were obtained as the side products.

**Table 1:** Reactions of K_2_ **1** with selected fluoro(hetero)arenes

Entry	Substrate	LiCl^*a*^	K_2_ **1** ^*b*^	Conditions	[BH(CN)_3_]^–^ ^*c*^	Major tricyanoborate anion(s) formed		Isolated yield
1	C_6_FH_5_	Yes	<1	80 °C, 2 d	25%	[1-{(NC)_3_B}-C_6_H_5_]^–^ (**B1**)	Sole isomer	45%
2	1,2-C_6_F_2_H_4_	Yes	<1	r.t., 16 h	9%	[1-{(NC)_3_B}-2-F-C_6_H_4_]^–^ (**B2a**)	Sole isomer	58%
3	1,3-C_6_F_2_H_4_	Yes	<1	r.t., 3 d	<5%	[1-{(NC)_3_B}-3-F-C_6_H_4_]^–^ (**B2b**)	Sole isomer	70%
4	1,4-C_6_F_2_H_4_	Yes	<1	75 °C, 30 h	28%	[1-{(NC)_3_B}-4-F-C_6_H_4_]^–^ (**B2c**) + **B2b** (6 : 4^*d*^)^*e*^		45%^*e*^
5	1,2,3-C_6_F_3_H_3_	Yes	<1	r.t., 3 d	<5%	[1-{(NC)_3_B}-2,3-F_2_-C_6_H_3_]^–^ (**B3a**) + [1-{(NC)_3_B}-2,6-F_2_-C_6_H_3_]^–^ (**B3b**) (4 : 1)		66%
6	1,2,4-C_6_F_3_H_3_	Yes	<1	r.t., 2 h	<5%	[1-{(NC)_3_B}-2,5-F_2_-C_6_H_3_]^–^ (**B3c**)	Sole isomer	76%
7	1,3,5-C_6_F_3_H_3_	Yes	<1	r.t., 16 h	<5%	[1-{(NC)_3_B}-3,5-F_2_-C_6_H_3_]^–^ (**B3d**)	Sole isomer	63%
8	1,2,3,4-C_6_F_4_H_2_	Yes	<1	r.t., <1 h	<5%	[1-{(NC)_3_B}-2,3,6-F_3_-C_6_H_2_]^–^ (**B4a**)	Sole isomer	77%
9	1,2,3,5-C_6_F_4_H_2_	Yes	<1	r.t., 2 h	<5%	[1-{(NC)_3_B}-2,3,5-F_3_-C_6_H_2_]^–^ (**B4b**)	Sole isomer	63%
10	1,2,3,5-C_6_F_4_H_2_	No	<1	75 °C, 30 h	10%	**B4b**	8% of other isomers	51%
11	1,2,4,5-C_6_F_4_H_2_	Yes	<1	r.t., 30 min	30%	[1-{(NC)_3_B}-2,4,5-F_3_-C_6_H_2_]^–^ (**B4c**)	Sole isomer	50%
12	1,2,4,5-C_6_F_4_H_2_	Yes	2	r.t., 16 h	n.d.	[1,4-{(NC)_3_B}_2_-2,5-F_2_-C_6_H_2_]^2–^ (**B4d**)	Sole isomer	42%^*f*^
13	C_6_F_5_H	Yes	<1	r.t., 10 min	10%	[1-{(NC)_3_B}-2,3,5,6-F_4_-C_6_H]^–^ (**B5**)	6% of one other isomer	62%
14	C_6_F_5_H	No	<1	r.t., 2 d	48%	**B5**	20% of other anions	39%
15	C_6_F_6_	No	<1	0 °C	—	[1-{(NC)_3_B}-C_6_F_5_]^–^ (**B6a**)^29^	Sole isomer	67% (ref. 30)
16	C_6_F_6_	No	2.2	Reflux, 20 h	—	[1,4-{(NC)_3_B}_2_-C_6_F_4_]^2–^ (**B6b**)	Sole isomer	74%
17	C_5_F_5_N	No	0.33	r.t., 12 h	—	[4-{(NC)_3_B}-C_5_F_4_N]^–^ (**Py1**)^*g*^	Other isomer(s), **Py2**, **Py3**	81%^*h*^
18	C_5_F_5_N	No	2	r.t., 4 d	—	[2,4-{(NC)_3_B}_2_-C_5_F_3_N]^2–^ (**Py2**)^*i*^	Other isomer(s), **Py3**	59%^*j*^
19	C_5_F_5_N	No	3	r.t., 4 d	—	[2,4,6-{(NC)_3_B}_3_-C_5_F_2_N]^3–^ (**Py3**)	<10% **Py1** and **Py2**	31%
20	C_12_F_10_	No	0.8	r.t., 2 h	—	[4-{(NC)_3_B}-C_12_F_9_]^–^ (**BP1**)	<20% of **BP2**	49%
21	C_12_F_10_	No	2.2	50 °C, 1 h	—	[4,4′-{(NC)_3_B}_2_-C_12_F_8_]^2–^ (**BP2**)	Sole isomer	90%
22	C_10_F_8_	No	0.5	r.t., 3 d	—	[2-{(NC)_3_B}-C_10_F_7_]^–^ (**N1**)	8% of other isomers^*k*^	55%
23	C_10_F_8_	No	2	60 °C, 16 h	—	[2,6-{(NC)_3_B}_2_-C_10_F_6_]^2–^ (**N2**)	Mixture of **N1**, **N2** and **N3** ^*l*^	24%
24	F_3_C–C_6_F_5_	No	<1	r.t., 3 h	—	[1-F_3_C-4-{(NC)_3_B}-C_6_F_4_]^–^ (**B7**)	Sole isomer	68%
25	Me–C_6_F_5_	No	<1	90 °C, 3 d	—	[1-Me-4-{(NC)_3_B}-C_6_F_4_]^–^ (**B8a**) + [1-Me-3-{(NC)_3_B}-C_6_F_4_]^–^ (**B8b**) (9 : 1)		n.d.
26	1-F_3_C-4-H-C_6_F_4_	No	<1	r.t., 2 h	75%	Unidentified borate anions		n.d.
27	1-F_3_C-6-H-C_6_F_4_	No	<1	r.t., 4 d	2%	[1-F_3_C-4-{(NC)_3_B}-2,3,5-F_3_-C_6_H]^–^ (**B9**)	7% of other isomers	78%
28	MeO–C_6_F_5_	No	<1	60 °C, 16 h	—	[1-MeO-4-{(NC)_3_B}-C_6_F_4_]^–^ (**B10a**) + [1-MeO-3-{(NC)_3_B}-C_6_F_4_]^–^ (**B10b**) (1 : 1)		82%
29	NC-C_6_F_5_	No	<1	r.t., <1 h	—	[1-NC-4-{(NC)_3_B}-C_6_F_4_]^–^ (**B11a**) + [1-NC-2-{(NC)_3_B}-C_6_F_4_]^–^ (**B11b**) + [B(CN)_4_]^–^ (2.5 : 1.5 : 1)^*m*^		n.d.
30	Cl–C_6_F_5_	No	<1	r.t., <1 h	—	[BCl(CN)_3_]^–^, [B_2_(CN)_6_]^2–^, …		n.d.
31	O_2_N–C_6_F_5_	No	<1	r.t., 16 h	—	[B_2_(CN)_6_]^2–^, …		n.d.

^*a*^Whether LiCl was added to the reaction mixture.

^*b*^Equivalents of K_2_ **1**.

^*c*^The percentage that was formed as a side product; [BH(CN)_3_]^–^ was removed during the work-up.

^*d*^The ratio **B2c** : **B2b** was 6 : 4 in the reaction mixture and 7 : 3 in the isolated material.

^*e*^11% of K[BH(CN)_3_].

^*f*^[Et_3_NH]^+^ salt.

^*g*^The internal yield was 75% **Py1**, 16% **Py2**, 8% another isomer (probably [2-{(NC)_3_B}-C_5_F_4_N]^–^), and 1% **Py3** and unknown tricyano(fluoropyridinyl)borate anions.

^*h*^Purity *ca.* 85% (^11^B/^19^F NMR); it contained 15% other tricyano(fluoropyridinyl)borates.

^*i*^The internal yield (^11^B/^19^F NMR) was 60% **Py2**, 30% **Py3**, and 10% another tricyano(fluoropyridinyl)borate anion.

^*j*^Purity *ca.* 75% (^11^B/^19^F NMR); it contained 15% K_3_ **Py3** and 10% another tricyano(fluoropyridinyl)borate.

^*k*^ **N2** : **N3** = 1.0 : 0.8 (**N3** = [2,7-{(NC)_3_B}_2_-C_10_F_6_]^2–^).

^*l*^The ratio of the reaction mixture: **N1** : **N2** : **N3** = 0.4 : 1.0 : 0.7; **N2** is hardly soluble and was obtained as a pure K^+^ salt.

^*m*^K[B(CN)_4_] was mostly removed *via* fractional precipitation.

An excess of K_2_ **1** yielded larger amounts of the dianion **Py2** and the trianion **Py3** and the reaction of perfluoropyridine with two equivalents of K_2_ **1** gave K_2_ **Py2** as the major product. Three equivalents of the potassium salt resulted in K_3_ **Py3** in 31% yield. A related, successive replacement of 1, 2 and 3 fluorine substituents of perfluoropyridine *via* nucleophilic replacement has been previously reported for the methoxy anion.^54^ In contrast, alkyl and aryl Grignard reagents were reported to result in only mono-substitutions in the *para* position of perfluoropyridine.^55^ The decreasing solubility of the potassium borates K**Py1**, K_2_ **Py2** and K_3_ **Py3** with the increasing charge of the anion enabled the enrichment of K_2_ **Py2** and the purification of K_3_ **Py3** by precipitation from the THF solutions *via* the slow addition of CH_2_Cl_2_. Pure K**Py1** and K_2_ **Py2** were obtained *via* crystallization. Single-crystals of K**Py1**, K_2_ **Py2**·OC(CH_3_)_2_ and K_3_ **Py3**·3THF·1.04 H_2_O were studied using X-ray diffraction (Fig. 1). Selected experimental bond distances of the three related anions **Py1**, **Py2** and **Py3** were compared to the calculated bond lengths in Table S3.†

### Monosubstitution of partly fluorinated benzenes with K_2_ **1**

**Fig. 2:**
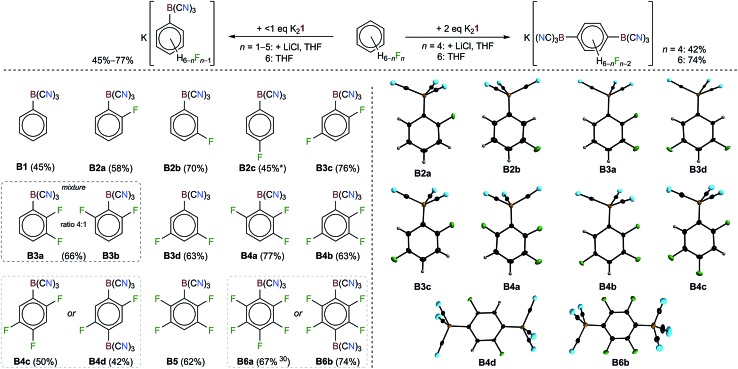
Reactions of K_2_ **1** with fluorinated benzenes (top). The main products; dashed boxes show the anions that were derived from the same arene. In the cases of C_6_F_6_ and 1,2,4,5-C_6_F_4_H_2_, the different anions were obtained from different entries (bottom left). The tricyanophenylborate anions in the crystal structures of their K^+^ (**B2a**, **B2b**, **B3a**, **B3c**, **B3d**, **B4a**, and **B4b**), [Et_4_N]^+^ (**B6b**), [Et_3_NH]^+^ (**B4d**) and [Me_3_NH]^+^ (**B4c**) salts (the displacement ellipsoids are at the 50% probability level, except for **B3a** where they are at 25%, and the H atoms are depicted with arbitrary radii) (bottom right).

In addition to the reactions of K_2_ **1** with hexafluorobenzene^30^ and pentafluoropyridine, K_2_ **1** was found to undergo C–F/C–B exchange reactions with all of the partly fluorinated benzenes C_6_F_6–*n*_H_*n*_ (*n* = 1–5) (Table 1 and Fig. 2). Unprecedentedly, the nucleophilic attack of **1** at almost all of the partially fluorinated benzenes was regio- and chemoselective. As expected for electron deficient partly fluorinated benzenes, deprotonation was observed for the strong base **1**, yielding [BH(CN)_3_]^–^ as a side product. The amount of [BH(CN)_3_]^–^ formed reflects the Brønsted acidity of the hydrogen atoms of the respective fluorobenzene.^56,57^ 1,2,4,5-tetrafluoro- and pentafluorobenzene gave the largest amounts of [BH(CN)_3_]^–^ with up to 48% for C_6_F_5_H (Table 1). The addition of LiCl at the start of the reaction of K_2_ **1** with C_6_F_5_H resulted in a reduction in the amount of [BH(CN)_3_]^–^ to 10% and an increased yield of [1-{(NC)_3_B}-2,3,5,6-F_4_-C_6_H]^–^ (**B5**) of 62%. Since the salts [(alkyl)_3_NH][BH(CN)_3_] (alkyl = Me, Et) are water soluble, purification was achieved *via* the precipitation or extraction of the respective trimethyl- or triethylammonium phenylborates from aqueous solutions. The ammonium salts were easily back-converted into K^+^ salts with K_2_CO_3_.

**Scheme 2:**
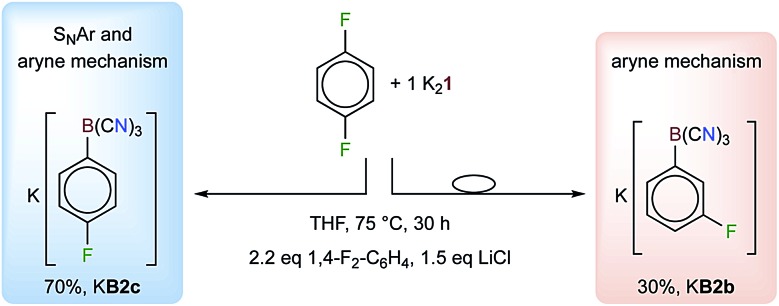
Reaction of 1,4-F_2_-C_6_H_4_ with K_2_ **1** in the presence of LiCl to give a mixture of K**B2** and K**B3** (ratio 7 : 3). The [BH(CN)_3_]^–^ that formed (25%) is not shown.

The regioselectivities of the C–F/C–B exchange reactions were found to be high, and in most cases one major isomer had formed (Table 1). Most of the new tricyanoborates were characterized using single-crystal X-ray diffraction (Fig. 2) and the details of the experimental and calculated bond parameters are summarized in Table S3.† The exchange of a fluorine with a tricyanoboryl group in the *para* position to a fluorine substituent is unfavoured. Replacement was found to be preferred for fluorine substituents in the *meta* position to one or two further fluorine substituents, which is typical for the S_N_Ar reactions of fluorobenzenes.^52,53^ 1,4-difluorobenzene gave a mixture of [1-{(NC)_3_B}-4-F-C_6_H_4_]^–^ (**B2c**) and [1-{(NC)_3_B}-3-F-C_6_H_4_]^–^ (**B2b**) in conjunction with 25% of [BH(CN)_3_]^–^ (Scheme 2). The formation of anion **B2b** is rationalized by an aryne mechanism that is similar to related reactions.^58^ In summary, the high regioselectivities that were observed show that an S_N_Ar mechanism dominates for the C–F/C–B exchange presented herein.

The reaction rate of the C–F/C–B exchange strongly depended on the degree of fluorination and therefore on the electron density of the aromatic ring system. C_6_F_6_ reacted within minutes at room temperature, whereas the conversion of C_6_F_5_H required two days. In the cases of mono-, di- and trifluorobenzenes, no reaction was observed with K_2_ **1** in THF even at 80 °C. Tetrafluorobenzenes showed some reactivity towards K_2_ **1** in THF depending on the substitution scheme. 1,2,3,5-tetrafluorobenzene reacted at 75 °C within 3 days. The reaction of the 1,2,4,5-isomer required 120 °C, yielding an inseparable brownish mixture. The addition of anhydrous LiCl was found to result in a tremendous increase in the reaction rate. For example, upon the addition of LiCl, a mixture of 1,2,4,5-tetrafluorobenzene and K_2_ **1** immediately became warm and the reaction was complete within minutes. Similarly, the reaction time of the conversion of C_6_F_5_H into **B5** was reduced from 2 days to 10 minutes. The shorter reaction time was accompanied by enhanced chemo- and regioselectivity (Table 1). The LiCl-induced reaction was successfully applied for all of the di- and trifluorobenzenes and C_6_FH_5_ (Fig. 1). The reactions of all three trifluorobenzene isomers and of 1,2- and 1,3-difluorobenzene were conducted at room temperature. Only for 1,4-F_2_-C_6_H_4_ and C_6_FH_5_ were higher temperatures necessary. Three different effects may be responsible for the faster reactions and the higher chemo- and regioselectivities in the presence of LiCl: (i) Li_2_ **1** is more soluble in THF than K_2_ **1**,^31^ which results in an enhanced availability of the dianion **1**; (ii) the high fluoride ion affinity of Li^+^ may lead to a Li···F interaction, a weakening of the C–F bond and a lowering of the activation barrier for the nucleophilic replacement; and (iii) a weak Li···B or Li···N interaction between Li^+^ and the boryl dianion **1** may influence the reactivity of **1**.

### Disubstitution of fluorinated benzenes with K_2_ **1**

C_6_F_6_ and 1,2,4,5-F_4_-C_6_H_2_ were reacted with an excess of K_2_ **1** to give salts of the dianions [1,4-{(NC)_3_B}_2_-C_6_F_4_]^2–^ (**B6b**) and [1,4-{(NC)_3_B}_2_-2,6-F_2_-C_6_H_2_]^2–^ (**B4d**) (Table 1 and Fig. 2). In the case of the reaction of 1,2,4,5-F_4_-C_6_H_2_, anhydrous LiCl was added to enhance the rate of the reaction. Both of the reactions were found to be highly regioselective and in accordance with an S_N_Ar mechanism. Therefore, the second C–F/C–B exchange gave the *para*-{B(CN)_3_}_2_ derivatives as the sole isomers.

**Fig. 3:**
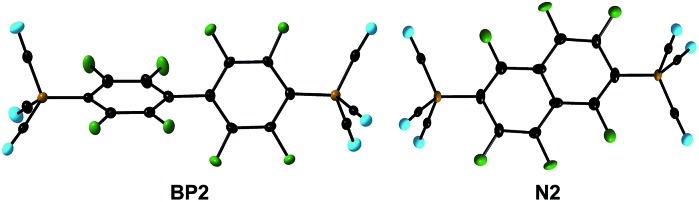
The bis(tricyanoborate) dianions **BP2** and **N2** in the crystal structures of their K^+^ salts (the displacement ellipsoids are at the 50% probability level).

In addition to the multiple C–F/C–B exchange reactions of C_5_F_5_N (Fig. 1), C_6_F_6_ and 1,2,4,5-F_4_-C_6_H_2_ (Fig. 2), perfluorobiphenyl and perfluoronaphthalene were successfully applied in related reactions with K_2_ **1**. Stoichiometric amounts or a slight excess of K_2_ **1** yielded K_2_[4,4′-{(NC)_3_B}_2_-C_12_F_8_] (K_2_ **BP2**) and K_2_[2,6-{(NC)_3_B}_2_-C_10_F_6_] (K_2_ **N2**), respectively. In contrast, an excess of the perfluorinated arene predominantly gave the corresponding monoanions [4-{(NC)_3_B}-C_12_F_9_]^–^ (**BP1**) and [2-{(NC)_3_B}-C_10_F_7_]^–^ (**N1**) (Table 1). The successive reactions of perfluorobiphenyl that gave **BP1** and **BP2** were fully regioselective. K_2_ **BP2** was isolated in an excellent yield of 90% and a single crystal was investigated using X-ray diffraction (Fig. 3 and Table S3†). A slightly lower but still high regioselectivity was found for the first replacement of fluorine with **1** in perfluoronaphthalene to give **N1**. The introduction of a second {B(CN)_3_} moiety resulted in a mixture of **N1**, **N2** and [2,7-{(NC)_3_B}_2_-C_10_F_6_]^2–^ (**N3**) with the ratio 0.4 : 1.0 : 0.7. The formation of **N2** as the major isomer agrees with the typical substitution scheme for the S_N_Ar reactions of perfluoronaphthalene.^53^

### Reactions of K_2_ **1** with fluorinated benzenes with functional groups

**Fig. 4:**
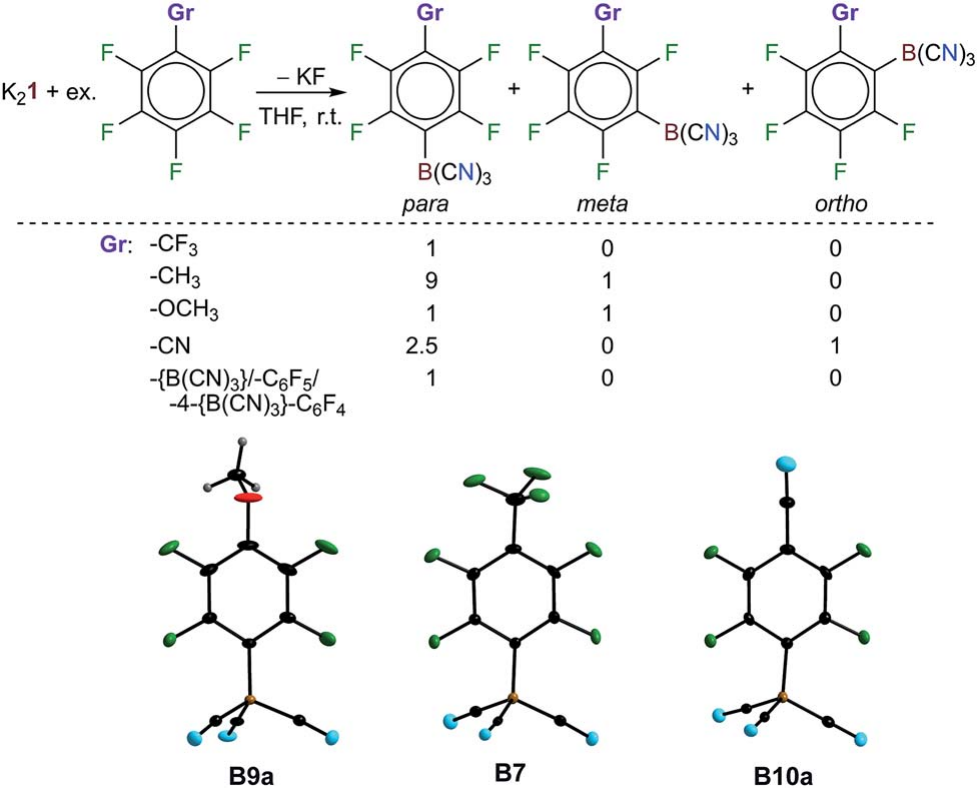
Reactions of K_2_ **1** with functionalized fluorinated benzenes. Borate anions **B9a**, **B7** and **B10a** in the crystal structures of their K^+^ salts (the displacement ellipsoids are at the 25% probability level, except for **B10a** where they are at 50%, and the H atoms are depicted with arbitrary radii).

The reactions described so far (*vide supra*) demonstrate the regioselectivity of the S_N_Ar reaction of fluorinated (hetero)arenes with K_2_ **1** and show the possibility of synthesising multiple charged tricyanoborate anions. The multiple C–F/C–B exchange reactions are the first examples of the transformations of functionalized fluorinated (hetero)arenes with **1**. A series of further selected reactions of polyfluorinated benzenes with a functional group bonded to the benzene ring have been studied (Fig. 4 and Table 1, entries 24–31).

Perfluorotoluene was found to give [1-F_3_C-4-{(NC)_3_B}-C_6_F_4_]^–^ (**B7**) as the sole isomer and K**B7** was isolated in 68% yield. 4-Me-C_6_F_5_ resulted in a mixture of the isomers [1-Me-4-{(NC)_3_B}-C_6_F_4_]^–^ (**B8a**) and [1-Me-3-{(NC)_3_B}-C_6_F_4_]^–^ (**B8b**) in a 9 : 1 ratio and pentafluoroanisole gave [1-MeO-4-{(NC)_3_B}-C_6_F_4_]^–^ (**B10a**) and [1-MeO-3-{(NC)_3_B}-C_6_F_4_]^–^ (**B10b**) in equal amounts. The decrease in the regioselectivity in the order F_3_C–C_6_F_5_, Me–C_6_F_5_ and MeO–C_6_F_5_ reflects the influence of the electronic properties of the –CF_3_, –Me and –OMe substituents on the reactivity of the corresponding fluoroarenes. The trifluoromethyl group is a strong electron withdrawing group, while the methoxy group is an electron donating group. Strong electron withdrawing groups are usually *para* and *ortho* directing. It is most likely that steric effects are the reason that the *ortho*-substituted product [1-F_3_C-2-{(NC)_3_B}-C_6_F_4_]^–^ was not observed. The reaction of K_2_ **1** with NC-C_6_F_5_, which contains the strong electron withdrawing cyano group that is sterically less demanding than the CF_3_ group, gave the *ortho*-substituted anion [1-NC-2-{(NC)_3_B}-C_6_F_4_]^–^ (**B11b**) together with [1-NC-4-{(NC)_3_B}-C_6_F_4_]^–^ (**B11a**) and [B(CN)_4_]^–^ in a ratio of 1.5 : 2.5 : 1. The formation of the tetracyanoborate anion is due to the nucleophilic attack at the carbon atom of the cyano group. The formation of [B(CN)_4_]^–^ starting from K_2_ **1** was reported previously, *e.g.* from reactions with (CN)_2_ and PhOCN.^31^ Single crystals of the potassium salts of [1-F_3_C-4-{(NC)_3_B}-C_6_F_4_]^–^ (**B7**), [1-MeO-4-{(NC)_3_B}-C_6_F_4_]^–^ (**B10a**) and [1-NC-4-{(NC)_3_B}-C_6_F_4_]^–^ (**B11a**) were characterized using diffraction experiments (Fig. 4 and Table S3†).

The deprotonation of 1-F_3_C-4-H-C_6_F_4_ to give [BH(CN)_3_]^–^ was found to be the most relevant reaction with K_2_ **1**, and other unidentified borate anions had formed. In contrast, 1-F_3_C-2-H-C_6_F_4_ and K_2_ **1** gave the [1-F_3_C-4-{(NC)_3_B}-2,3,5-F_3_-C_6_H]^–^ anion (**B9**) in 78% yield and only 2% of [BH(CN)_3_]^–^ was observed (Table 1).

The reactions of K_2_ **1** with Cl–C_6_F_5_ and O_2_N–C_6_F_5_ gave complex product mixtures, and the hexacyanoborate dianion [B_2_(CN)_6_]^2–^ (ref. 59) was identified. Additionally, the tricyanohaloborate anion [BCl(CN)_3_]^–^ (ref. 60) formed from Cl–C_6_F_5_ (Table 1).

## Conclusions

Boron-centred nucleophiles are an emerging class of compounds and are of growing importance for synthetic chemistry because they provide a very convenient entry to boron-functionalized compounds and materials.^1–28^ The unprecedented, straightforward, regioselective and transition-metal-free introduction of one, two or even three {B(CN)_3_}^–^ units into fluoro(hetero)arenes starting from readily accessible K_2_ **1** ^30–32^ opens unique and convenient access to single and multiple charged anions with tricyanoborate moieties. The observed regioselectivities for the C–F/C–B exchange reactions are almost all in accordance with an S_N_Ar mechanism, which showed the boron-centred nucleophilic character of the dianion **1**, as previously observed for the formation of [B_2_(CN)_6_]^2–^ starting from K_2_ **1** and K[BF(CN)_3_].^59^ So, an alternative radical reaction pathway is highly unlikely, although this type of reactivity was observed for other boron-centred nucleophiles.^61,62^ Furthermore, the syntheses highlight the value of K_2_ **1** for the general preparation of a wealth of tricyanoborate anions. Since the salts of the tricyanoborate anions such as the ones described herein are promising building blocks for materials science, for example as components of ionic liquids that are used in electrochemical devices,^63^ convenient and high yield syntheses are necessary to facilitate applications.
