# Supplementary material for: Borylation of fluorinated arenes using the boron-centred nucleophile B(CN)3 2– – a unique entry to aryltricyanoborates
Source: Chem Sci. 2017 Jun 26;8(9):5962–8. doi: 10.1039/c7sc02249b (PMC5620525; doi:10.1039/c7sc02249b)
Supplement: Supplementary file 1 [file SC-008-C7SC02249B-s001.pdf]

Electronic Supplementary Material (ESI) for Chemical Science.
This journal is © The Royal Society of Chemistry 2017

Supporting information

Borylation of Fluorinated Arenes with the Boron-centred Nucleophile $B(CN)_3^{2-}$ – a Unique and Convenient Access to Aryltricyanoborates

Johannes Landmann,[a] *Philipp T. Hennig,*[a] *Nikolai V. Ignat'ev,*[b] *and Maik Finze**[a]

Affiliations

[a] Institut für Anorganische Chemie, Institut für nachhaltige Chemie & Katalyse mit Bor (ICB), Julius-Maximilians Universität Würzburg, Am Hubland, 97074 Würzburg, Germany. Web: https://go.uniwue.de/finze-group. E-mail: maik.finze@uni-wuerzburg.de.

[b] Consultant, Merck KGaA, Frankfurter Straße 250, 64293 Darmstadt, Germany.

*Correspondence to: maik.finze@uni-wuerzburg.de.

Table of Contents

1. General Synthetic Aspects S3
2. Analytical Instruments and Details S3
3. Chemicals S4
4. Syntheses S4
5. Table of Experimental and Calculated Chemical Shifts S24
6. Crystal Structure Determinations S27
7. Experimental and Calculated Bond Lengths S30
8. References S32

1. General Synthetic Aspects

Reactions involving air-sensitive compounds were performed either in round bottom flasks or in glass tubes equipped with valves with PTFE stems (Rettberg, Göttingen and Young, London) under argon using standard Schlenk line techniques.

2. Analytical Instruments and Details, DFT Calculations

^{1}H, ^{11}B and ^{13}C NMR spectra were recorded at 25 °C in $(CD_3)_2CO$ on a Bruker Avance 500 or on a Bruker Avance III HD 300 spectrometer. NMR signals were referenced against TMS (^{1}H and ^{13}C; $\Xi(^{13}C)$ = 25.145020 MHz) and $BF_3{\cdot}OEt_2$ in $CDCl_3$ ($\Xi(^{11}B)$ = 32.083974 MHz).[1] Chemical shifts were calibrated against the residual solvent signal and the solvent signal, respectively ($\delta(^1H)$: $(CD_2H)(CD_3)CO$ 2.05 ppm; $\delta(^{13}C)$: $(CD_3)_2CO$ 206.26 and 29.84 ppm.[2] The labeling of the nuclei according to Figure S1.

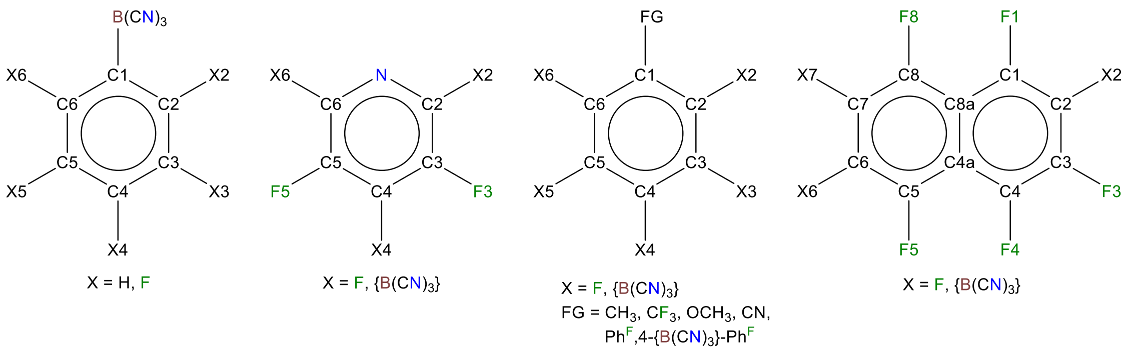

Figure S1. Labeling of the nuclei and positions of the borate anions.

The assignment of the NMR signals is aided by chemical shifts derived from DFT calculations.[3] DFT-GIAO[4] NMR shielding constants $\sigma(^{11}B)$, $\sigma(^{13}C)$, $\sigma(^1H)$ and $\sigma(^{19}F)$ were calculated at the B3LYP/6-311++G(2d,p) level of theory using the geometries computed at the B3LYP/6-311++G(d,p) level of theory. The ^{11}B, ^{13}C, ^{1}H and ^{19}F NMR shielding constants were calibrated to the respective chemical shift scale $\delta(^{11}B)$, $\delta(^{13}C)$, $\delta(^1H)$ and $\delta(^{19}F)$ using predictions on diborane(6), Me_4Si, and $CFCl_3$ with chemical shifts of 16.6 ppm for B_2H_6[5] and 0 ppm for Me_4Si and $CFCl_3$.[6] All calculations were performed using the Gaussian09 program suite.[7]

Elemental analyses (C, H, N) were performed either with a Euro EA3000 instrument (HEKA-Tech, Germany) or with an Elementar Vario MICRO cube instrument (Elementar Analysensysteme, Germany).

3. Chemicals

All standard chemicals were obtained from commercial sources und used without further purification. Solvents were dried according to standard protocols[8] and stored in flasks equipped with valves with PTFE stems (Young, London) under an argon atmosphere. $K_2B(CN)_3$ (K_2**1**) was synthesized according to known procedures.[9]

4. Syntheses

General procedures for the syntheses of the borates.

If not stated otherwise, the synthesis of the borate anions were performed according to method A (without LiCl) or method B (in the presence of LiCl). The work-up procedure described for method B was applied for the removal of $[BH(CN)_3]^-$, in general.

Method A: $K_2B(CN)_3$ (K_2**1**) was suspended in THF (5–8 mL). The fluoroarene was added in a single portion at room temperature either as neat compound (liquid fluoroarenes) or as a solution in THF (solid fluoroarenes) using a syringe. The end of the reaction was either evident from decolorization of **1** (yellow compound) or by ^{19}F and ^{11}B NMR spectroscopy. For the NMR spectroscopic control experiments, a small sample of the suspension was taken while stirring and dissolved in H_2O to give a clear solution. Residual solid **1** was converted into $[BH(CN)_3]^-$ and the hydridoborate anion was detected by ^{11}B NMR spectroscopy. If necessary, the reaction mixture was stirred at elevated temperatures.

All solid material that had formed (mainly KF) was separated by filtration through a plug of Celite. The Celite plug was washed with THF (3 × 5–10 mL), the solvent of the combined THF layers was removed, and the residue was dried in a vacuum (removal of excess arene). The residue was dissolved in THF (5 mL) again and the product was precipitated by slow addition of CH_2Cl_2. Fractional precipitated allowed the separation of by- and/or side products. The off-white to colourless solids were dried in a vacuum.

Method B: In contrast to method A, LiCl (ca. 1.5 equivalents) was added to the reaction mixture. After completion of the reaction (see method A) the combined THF layers were dried in a vacuum. The residue was dissolved in slightly acidified H_2O (ca. 20 mL) and an aqueous solution of an excess of $[Me_3NH]Cl$ or $[Et_3NH]Cl$ was slowly added, which resulted in the precipitation of the respective organic salt either as a solid that was filtered off or the formation of an oily material. In the latter case the mixture was extracted with CH_2Cl_2 (3 × 15

mL) and the combined organic layers were washed with H_2O (5 × 2 mL). After removal of all volatiles the oily remainder of the CH_2Cl_2 phases, H_2O (25 mL) and an excess of K_2CO_3 was added in several portions. After dissolution, the mixture was concentrated under reduced pressure until K_2CO_3 began to precipitate. The mixture was extracted with THF (3 × 20 mL), the combined organic layers were dried with K_2CO_3, filtered and concentrated to a volume of ~5 mL. CH_2Cl_2 (20 mL) was added and an off-white to colourless precipitate formed that was collected by filtration and dried in a vacuum.

K[1-{(NC)$_3$B}-C$_6$H$_5$] (KB1): Method B was employed for the preparation of K**B1**, using fluorobenzene (ca. 0.5 mL, 5.4 mmol), K_2**1** (200 mg, 1.20 mmol) and LiCl (150 mg, 3.54 mmol). The reaction mixture was heated to 80 °C for 2 days. Yield: 110 mg (0.536 mmol, 45%). ^{11}B NMR (160.5 MHz, $(CD_3)_2CO$): $\delta = -28.9$ ppm (s, 1B). ^{1}H NMR (500.1 MHz, $(CD_3)_2CO$): $\delta = 7.54$–7.49 (m, 2H, H2/6), 7.28–7.24 (m, 2H, H3/5), 7.21–7.16 ppm (m, 1H, H4). ^{1}H{^{11}B} NMR (500.1 MHz, $(CD_3)_2CO$): $\delta =$ 7.54–7.49 (m, 2H, H2/6), 7.28–7.24 (m, 2H, H3/5), 7.21–7.16 ppm (m, 1H, H4). ^{13}C{^{1}H} NMR (125.8 MHz, $(CD_3)_2CO$): $\delta = 140.6$ (q, 1C, $^{1}J(^{13}C,^{11}B) = 56$ Hz, C1), 133.3 (s, 2C, C2/6), 129.7 (q, 3C, $^{1}J(^{13}C,^{11}B) = 64$ Hz, CN), 128.3 (s, 2C, C3/5), 127.2 ppm (s, 1C, C4). Elemental analysis: calculated (%) for $C_9H_5BKN_3$, C 52.71, H 2.46, N 20.49; found, C 51.89, H 2.55, N 20.77. (−)-ESI-MS, m/z, $[(C_6H_5)B(CN)_3]^-$: calculated 166.06 (100.0%), 165.06 (24.8%), 167.06 (9.7%); found 166.06 (100.0%), 165.06 (25.5%), 167.06 (9.7%).

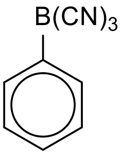

B1

K[1-{(NC)$_3$B}-2-F-C$_6$H$_4$] (KB2a): Method B was employed for the preparation of K**B2a**, using 1,2-difluorobenzene (0.5 mL, 5.1 mmol), K_2**1** (350 mg, 2.10 mmol) and LiCl (150 mg, 3.54 mmol). The reaction mixture was stirred at room temperature overnight. Yield: 270 mg (1.21 mmol, 58%). ^{11}B NMR (160.5 MHz, $(CD_3)_2CO$): $\delta = -30.8$ ppm (s, 1B). ^{11}B{^{1}H} NMR (160.5 MHz, $(CD_3)_2CO$): $\delta = -30.8$ ppm (d, 1B, $^{3}J(^{19}F,^{11}B) \approx 8$ Hz). ^{1}H NMR (500.1 MHz, $(CD_3)_2CO$): $\delta = 7.54$ (s, br, 1H, H6), 7.30–7.25 (m, 1H, H4), 7.09 (dddd, 1H, $J = 7.4$ Hz (2×), 1.1 Hz, 0.4 Hz, H5), 6.96 ppm (ddd, br, 1H, $J = 9.4$ Hz, 8.2 Hz, 1.1 Hz, H3). ^{1}H{^{11}B} NMR (500.1 MHz, $(CD_3)_2CO$): $\delta = 7.54$ (dddd, 1H, $J = 7.4$ Hz, 6.8 Hz, 1.9 Hz, 0.3 Hz, H6) [other signals as stated for the ^{1}H NMR spectrum]. ^{19}F NMR (470.6 MHz, $(CD_3)_2CO$): $\delta = -105.1$ ppm (s, 1F). ^{13}C{^{1}H} NMR (125.8 MHz, $(CD_3)_2CO$): $\delta = 166.3$ (d, 1C, $^{1}J(^{19}F,^{13}C) = 243$ Hz, C2), 135.4 (d, 1C, $^{3}J(^{19}F,^{13}C) = 8.8$ Hz, C6), 130.0 (d, 1C, $^{3}J(^{19}F,^{13}C) = 8.3$ Hz, C4), 128.5 (q, 3C, $^{1}J(^{13}C,^{11}B) = 65$ Hz, CN),

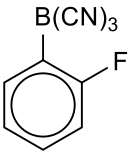

B2a

126.2 (qd, 1C, $^{1}J(^{13}C,^{11}B)$ = 56 Hz, $^{2}J(^{19}F,^{13}C)$ ≈ 23 Hz, C1), 124.4 (s, 1C, C5), 115.1 ppm (d, 1C, $^{2}J(^{19}F,^{13}C)$ ≈ 24 Hz, C3). $^{13}C\{^{19}F\}$ NMR (125.8 MHz, $(CD_3)_2CO$): δ = 166.3 (dddd, 1C, $J(^{13}C,^{1}H)$ = 11 Hz (2×), 4.5 Hz, 1.7 Hz, C2), 135.4 (ddd, 1C, $^{1}J(^{13}C,^{1}H)$ = 159 Hz, $J(^{13}C,^{1}H)$ = 8.7 Hz, 2.1 Hz, C6), 130.7–129.3 (m, 1C, C4), 128.5 (q, 3C, $^{1}J(^{13}C,^{11}B)$ = 65 Hz, CN), 126.2 (q, 1C, $^{1}J(^{13}C,^{11}B)$ = 56 Hz, C1), 124.4 (dm, 1C, $^{1}J(^{13}C,^{1}H)$ = 159 Hz, C5), 115.1 ppm (dd, 1C, $^{1}J(^{13}C,^{1}H)$ = 161 Hz, $^{2}J(^{13}C,^{1}H)$ = 8.1 Hz, C3). Elemental analysis: calculated (%) for $C_9H_4BFKN_3$, C 48.46, H 1.81, N 18.84; found, C 48.17, H 1.62, N 18.72. (−)-ESI-MS, m/z, $[(C_6FH_4)B(CN)_3]^-$: calculated 184.05 (100.0%), 183.05 (24.8%), 185.05 (9.7%); found 184.05 (100.0%), 183.05 (25.2%), 185.05 (9.6%). Crystals of K**B2a** suitable for an X-ray diffraction study were obtained from an acetone solution by slow evaporation of the solvent.

K[1-{(NC)$_3$B}-3-F-C$_6$H$_4$] (K**B2b**): Method B was employed for the preparation of K**B2b**, using 1,3-difluorobenzene (0.4 mL, 4.1 mmol), K_2**1** (300 mg, 1.80 mmol) and LiCl (115 mg, 2.71 mmol). The reaction mixture was stirred at room temperature for 3 days. Yield: 280 mg (1.26 mmol, 70%). ^{11}B NMR (160.5 MHz, $(CD_3)_2CO$): δ = −29.1 ppm (s, 1B). ^{1}H NMR (500.1 MHz, $(CD_3)_2CO$): δ = 7.36–7.28 (m, 2H), 7.19 (d, br, 1H, $^{2}J(^{19}F,^{1}H)$ ≈ 10 Hz), 6.97–6.92 ppm (m, 1H) [A definite assignment even with the aid of calculated chemical shifts was not possible]. ^{19}F NMR (470.6 MHz, $(CD_3)_2CO$): δ = −116.1 ppm (m, 1F). $^{13}C\{^{1}H\}$ NMR (125.8 MHz, $(CD_3)_2CO$): δ = 163.4 (d, 1C, $^{1}J(^{19}F,^{13}C)$ = 244 Hz, C3), 144.1 (q, 1C, $^{1}J(^{13}C,^{11}B)$ ≈ 56 Hz, C1), 130.2 (d, 1C, $^{3}J(^{19}F,^{13}C)$ = 7.4 Hz, C5), 129.1 (d, 1C, $^{4}J(^{19}F,^{13}C)$ = 2.7 Hz, C6), 129.1 (q, 3C, $^{1}J(^{13}C,^{11}B)$ ≈ 64 Hz, CN), 119.3 (d, 1C, $^{2}J(^{19}F,^{13}C)$ = 19.3 Hz, C2), 113.9 ppm (d, 1C, $^{2}J(^{19}F,^{13}C)$ = 21.1 Hz, C4). $^{13}C\{^{19}F\}$ NMR (125.8 MHz, $(CD_3)_2CO$): δ = 163.4 (s, 1C, C3), 144.1 (q, 1C, $^{1}J(^{13}C,^{11}B)$ ≈ 56 Hz, C1), 130.2 (d, 1C, $^{1}J(^{13}C,^{1}H)$ = 161 Hz, C5), 129.1 (ddd, 1C, $^{1}J(^{13}C,^{1}H)$ = 159 Hz, $^{3}J(^{13}C,^{1}H)$ = 7.7 Hz (2×), C6), 129.1 (q, 3C, $^{1}J(^{13}C,^{11}B)$ ≈ 64 Hz, CN), 119.3 (dm, 1C, $^{1}J(^{13}C,^{1}H)$ = 159 Hz, C2), 113.9 ppm (dm, 1C, $^{1}J(^{13}C,^{1}H)$ = 162 Hz, C4). Elemental analysis: calculated (%) for $C_9H_4BFKN_3$, C 48.46, H 1.81, N 18.84; found, C 47.90, H 1.73, N 18.51. (−)-ESI-MS, m/z, $[(C_6FH_4)B(CN)_3]^-$: calculated 184.05 (100.0%), 183.05 (24.8%), 185.05 (9.7%); found 184.05 (100.0%), 183.05 (24.9%), 185.05 (9.4%). Crystals of K**B2b** suitable for an X-ray diffraction study were obtained from an *iso*propanol solution by slow evaporation of the solvent.

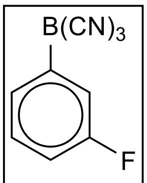

B2b

K[1-{(NC)$_3$B}-4-F-C$_6$H$_4$] (K**B2c**): Method B was employed for the preparation of K**B2c**, using 1,4-difluorobenzene (0.4 mL, 3.9 mmol), K$_2$**1** (300 mg, 1.80 mmol) and LiCl (115 mg, 2.71 mmol). The reaction mixture was stirred at 75 °C for 30 hrs. Yield: 180 mg (0.807 mmol, 45%). The product is a mixture of K**B2c** and K**B2b** (ratio 7:3) and additionally contained 11% K[BH(CN)$_3$]. The NMR spectroscopic data for K**B2b** match with those given above. NMR data of K**B2c**: ^{11}B NMR (160.5 MHz, (CD$_3$)$_2$CO): δ = −29.1 ppm (s, 1B). ^{1}H NMR (500.1 MHz, (CD$_3$)$_2$CO): δ = 7.50 (dd, br, 2H, $^{3}J(^{1}H,^{1}H)$ ≈ 8.6 Hz, $^{4}J(^{19}F,^{1}H)$ ≈ 6.1 Hz, H2/6), 7.01 ppm (ddd, 2H, $^{3}J(^{19}F,^{1}H)$ = 9.5 Hz, $^{3}J(^{1}H,^{1}H)$ = 8.6 Hz, H3/5). ^{19}F NMR (470.6 MHz, (CD$_3$)$_2$CO): δ = −118.6 ppm (tt, 1F, $^{3}J(^{19}F,^{1}H)$ = 9.4 Hz, $^{4}J(^{19}F,^{1}H)$ = 6.1 Hz). ^{13}C{^{1}H} NMR (125.8 MHz, (CD$_3$)$_2$CO): δ = 162.9 (d, 1C, $^{1}J(^{19}F,^{13}C)$ = 243 Hz, C4), 136.5 (q, 1C, $^{1}J(^{13}C,^{11}B)$ ≈ 56 Hz, C1), 135.0 (d, 2C, $^{3}J(^{19}F,^{13}C)$ = 7.4 Hz, C2/6), 129.5 (q, 3C, $^{1}J(^{13}C,^{11}B)$ ≈ 64 Hz, CN), 115.0 ppm (d, 2C, $^{2}J(^{19}F,^{13}C)$ = 20.0 Hz, C3/5). ^{13}C{^{19}F} NMR (125.8 MHz, (CD$_3$)$_2$CO): δ = 162.9 (tt, 1C, $^{2}J(^{13}C,^{1}H)$ = 10.9 Hz, $^{3}J(^{13}C,^{1}H)$ = 4.7 Hz, C4), 136.5 (m, 1C, C1), 135.0 (dd, 2C, $^{1}J(^{13}C,^{1}H)$ = 159 Hz, $^{n}J(^{13}C,^{1}H)$ = 8.9 Hz, C2/6), 129.5 (q, 3C, $^{1}J(^{13}C,^{11}B)$ ≈ 64 Hz, CN), 115.0 ppm (d, 2C, $^{1}J(^{13}C,^{1}H)$ = 162 Hz, C3/5). (−)-ESI-MS, m/z, [(C$_6$FH$_4$)B(CN)$_3$]$^-$: calculated 184.05 (100.0%), 183.05 (24.8%), 185.05 (9.7%); found 184.05 (100.0%), 183.05 (25.2%), 185.05 (9.4%).

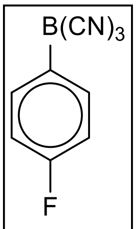

B2c

K[1-{(NC)$_3$B}-2,3-F$_2$-C$_6$H$_3$] (K**B3a**): Method B was employed for the preparation of K**B3a**, using 1,2,3-trifluorobenzene (0.4 mL, 3.9 mmol), K$_2$**1** (300 mg, 1.80 mmol) and LiCl (115 mg, 2.71 mmol). The reaction mixture was stirred at room temperature for 3 days. Yield: 285 mg (1.18 mmol, 66%). The product is a mixture of K**B3a** and K[1-{(NC)$_3$B}-2,6-F$_2$-C$_6$H$_3$] (K**B3b**) (ratio 4:1). NMR spectroscopic data of the main product K**B3a**: ^{11}B NMR (160.5 MHz, (CD$_3$)$_2$CO): δ = −31.0 ppm (s, br, 1B). ^{11}B{^{1}H} NMR (160.5 MHz, (CD$_3$)$_2$CO): δ = −31.0 ppm (dd, 1B, $^{3}J(^{19}F,^{11}B)$ = 8.4 Hz, $^{4}J(^{19}F,^{11}B)$ = 3.5 Hz). ^{1}H NMR (500.1 MHz, (CD$_3$)$_2$CO): δ = 7.34–7.27 (m, br, 1H, H6), 7.20–7.06 ppm (m, 2H, H4/5). ^{19}F NMR (470.6 MHz, (CD$_3$)$_2$CO): δ = −132.5 (m, 1F, F2), −142.3 ppm (m, 1F, F3). ^{13}C{^{1}H} NMR (125.8 MHz, (CD$_3$)$_2$CO): δ = 153.5 (dd, 1C, $^{1}J(^{19}F,^{13}C)$ = 244 Hz, $^{2}J(^{19}F,^{13}C)$ = 11.3 Hz, C2), 151.1 (dd, br, 1C, $^{1}J(^{19}F,^{13}C)$ = 246 Hz, $^{2}J(^{19}F,^{13}C)$ = 14.9 Hz, C3), 130.2 (dd, 1C, $J(^{19}F,^{13}C)$ = 7.6 Hz, 3.6 Hz, C6), 129.8 (qd, 1C, overlapped, C1), 128.1 (q, 3C, $^{1}J(^{13}C,^{11}B)$ ≈ 66 Hz, CN), 125.0 (s, br, 1C, C5), 116.9 ppm (d, 1C, $^{2}J(^{19}F,^{13}C)$ = 17.2 Hz, C4). ^{13}C{^{19}F}

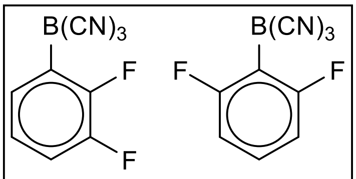

B3a **B3b**

NMR (125.8 MHz, $(CD_3)_2CO$): δ = 153.5 (dd, 1C, $^{3/4}J(^{13}C,^{1}H)$ = 11.9 Hz, 7.2 Hz, C2), 151.1 (s, br, 1C, C3), 130.2 (dd, 1C, $^{1}J(^{13}C,^{1}H)$ = 162 Hz, $^{2}J(^{13}C,^{1}H)$ ≈ 9 Hz, C6), 129.8 (q, 1C, overlapped, C1), 128.1 (q, 3C, $^{1}J(^{13}C,^{11}B)$ ≈ 66 Hz, CN), 125.0 (d, 1C, $^{1}J(^{13}C,^{1}H)$ = 163 Hz, C5), 116.9 ppm (dd, 1C, $^{1}J(^{13}C,^{1}H)$ = 164 Hz, $^{2}J(^{13}C,^{1}H)$ ≈ 9.5 Hz, C4). NMR spectroscopic data of the side product K**B3b**: ^{11}B NMR (160.5 MHz, $(CD_3)_2CO$): δ = −33.6 ppm (t, 1B $^{3}J(^{19}F,^{11}B)$ = 7.8 Hz). ^{1}H NMR (500.1 MHz, $(CD_3)_2CO$): δ = 7.31–7.25 (m, 1H, H4), 6.86–6.80 ppm (m, 2H, H3/5). ^{19}F NMR (470.6 MHz, $(CD_3)_2CO$): δ = −101.4 ppm (m, 2F, F2/6). $^{13}C\{^{1}H\}$ NMR (125.8 MHz, $(CD_3)_2CO$): δ = 166.1 (dd, 2C, $^{1}J(^{19}F,^{13}C)$ = 246 Hz, $^{2}J(^{19}F,^{13}C)$ = 13.0 Hz, C2/6), 130.5 (t, 1C, $^{3}J(^{19}F,^{13}C)$ = 10.8 Hz, C4), 128.2 (q, 3C, $^{1}J(^{13}C,^{11}B)$ ≈ 66 Hz, CN), 111.6 ppm (m, 2C, C3/5) [the signal of the C1 atom was not identified]. $^{13}C\{^{19}F\}$ NMR (125.8 MHz, $(CD_3)_2CO$): δ = 166.1 (d, 2C, $^{2}J(^{13}C,^{1}H)$ = 12.6 Hz, C2/6), 130.5 (d, 1C, $^{1}J(^{13}C,^{1}H)$ = 164 Hz, C4), 128.2 (q, 3C, $^{1}J(^{13}C,^{11}B)$ ≈ 66 Hz, CN), 111.6 (dd, 2C, $^{1}J(^{13}C,^{1}H)$ = 164 Hz, $^{2}J(^{13}C,^{1}H)$ = 7.8 Hz, C3/5) [the signal of the C1 atom was not identified]. Elemental analysis: calculated (%) for $C_9H_3BF_2KN_3$, C 44.85, H 1.25, N 17.43; found, C 44.89, H 1.22, N 17.42. (−)-ESI-MS, m/z, $[(C_6F_2H_3)B(CN)_3]^-$: calculated 202.04 (100.0%), 201.04 (24.8%), 203.04 (9.7%); found 202.04 (100.0%), 201.04 (24.5%), 203.04 (9.8%). Further purification of K**B3a** was achieved by crystallization from a solution in ethyl acetate by slow evaporation of the solvent. The single crystals obtained, were suitable for an X-ray diffraction study.

$K[1\text{-}\{(NC)_3B\}\text{-}2,5\text{-}F_2\text{-}C_6H_3]$ (K**B3c**): Method B was employed for the preparation of K**B3c**, using 1,2,4-trifluorobenzene (0.4 mL, 3.8 mmol), K_2**1** (300 mg, 1.80 mmol) and LiCl (115 mg, 2.71 mmol). The reaction mixture was stirred at room temperature for 2 hrs. Yield: 330 mg (1.37 mmol, 76%). ^{11}B NMR (160.5 MHz, $(CD_3)_2CO$): δ = −31.0 ppm (s, 1B). ^{1}H NMR (500.1 MHz, $(CD_3)_2CO$): δ = 7.20 (d, br, 1H, H6), 7.04–6.98 ppm (m, 2H, H3/4). $^{1}H\{^{11}B\}$ NMR (500.1 MHz, $(CD_3)_2CO$): δ = 7.22–7.18 (m, 1H, H6), 7.04–6.98 ppm (m, 2H, H3/4). ^{19}F NMR (470.6 MHz, $(CD_3)_2CO$): δ = −111.7 (s, br, 1F, F2), −122.1 (m, 1F, F5). $^{13}C\{^{1}H\}$ NMR (125.8 MHz, $(CD_3)_2CO$): δ = 162.1 (dd, 1C, $^{1}J(^{19}F,^{13}C)$ = 239 Hz, $^{4}J(^{19}F,^{13}C)$ = 1.9 Hz, C2), 159.4 (d, br, 1C, $^{1}J(^{19}F,^{13}C)$ = 240 Hz, C5), 128.8 (qd, 1C, $^{1}J(^{13}C,^{11}B)$ ≈ 56 Hz, $^{2}J(^{19}F,^{13}C)$ ≈ 25 Hz, C1), 128.0 (q, 3C, $^{1}J(^{13}C,^{11}B)$ = 66 Hz, CN), 120.9 (dd, 1C, $^{2}J(^{19}F,^{13}C)$ = 23.0 Hz, $^{3}J(^{19}F,^{13}C)$ = 9.5 Hz, C6), 116.6 (dd, 1C, $^{2}J(^{19}F,^{13}C)$ = 27.7 Hz, $^{3}J(^{19}F,^{13}C)$ = 8.3 Hz, C3), 115.9 ppm (dd, 1C, $^{2}J(^{19}F,^{13}C)$ = 24.4 Hz, $^{3}J(^{19}F,^{13}C)$ = 9.1 Hz, C4). $^{13}C\{^{19}F\}$ NMR (125.8 MHz, $(CD_3)_2CO$): δ = 162.3–162.0 (m, 1C, C2), 159.4 (s, br, 1C, C5), 128.8 (q, 1C, $^{1}J(^{13}C,^{11}B)$ ≈ 57 Hz, C1), 128.0

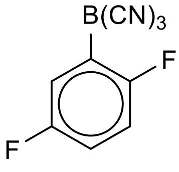

B3c

(q, 3C, $^{1}J(^{13}C,^{11}B)$ = 66 Hz, CN), 120.9 (d, 1C, $^{1}J(^{13}C,^{1}H)$ = 162 Hz, C6), 116.6 (d, 1C, $^{1}J(^{13}C,^{1}H)$ = 165 Hz, C3), 115.9 ppm (dd, 1C, $^{1}J(^{13}C,^{1}H)$ = 166 Hz, $^{3}J(^{13}C,^{1}H) \approx 5$ Hz, C4). Elemental analysis: calculated (%) for $C_9H_3BF_2KN_3$, C 44.85, H 1.25, N 17.43; found, C 44.15, H 1.27, N 16.52. (−)-ESI-MS, m/z, $[(C_6F_2H_3)B(CN)_3]^-$: calculated 202.04 (100.0%), 201.04 (24.8%), 203.04 (9.7%); found 202.04 (100.0%), 201.04 (25.3%), 203.04 (9.5%). Crystals of K**B3c** suitable for an X-ray diffraction study were obtained from an aqueous solution by slow evaporation of the solvent.

K[1-{(NC)$_3$B}-3,5-F$_2$-C$_6$H$_3$] (K**B3d**): <u>Method B</u> was employed for the preparation of K**B3d**,

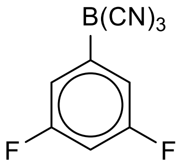

B3d

using 1,3,5-trifluorobenzene (0.3 mL, 2.9 mmol), K_2**1** (300 mg, 1.80 mmol) and LiCl (115 mg, 2.71 mmol). The reaction mixture was stirred at room temperature overnight. Yield: 275 mg (1.14 mmol, 63%). ^{11}B NMR (160.5 MHz, $(CD_3)_2CO$): $\delta = -29.3$ ppm (s, 1B). ^{1}H NMR (500.1 MHz, $(CD_3)_2CO$): $\delta = 7.04$ (m, 2H, H2/6), 6.81 ppm (tt, 1H, $^{3}J(^{19}F,^{1}H)$ = 9.3 Hz, $^{4}J(^{1}H,^{1}H)$ = 2.4 Hz, H4). ^{19}F NMR (470.6 MHz, $(CD_3)_2CO$): $\delta = -112.7$ ppm (m, 2F). $^{13}C\{^{1}H\}$ NMR (125.8 MHz, $(CD_3)_2CO$): $\delta = 163.6$ (d, 2C, $^{1}J(^{19}F,^{13}C)$ = 248 Hz, C3/5), 146.4 (q, 1C, $^{1}J(^{13}C,^{11}B)$ = 56 Hz, C1), 128.6 (q, 3C, $^{1}J(^{13}C,^{11}B)$ = 65 Hz, CN), 115.4 (m, 2C, C2/6), 102.4 (t, 1C, $^{2}J(^{19}F,^{13}C)$ = 25.6 Hz, C4). $^{13}C\{^{19}F\}$ NMR (125.8 MHz, $(CD_3)_2CO$): $\delta = 163.6$ (s, 2C, C3/5), 146.4 (q, 1C, $^{1}J(^{13}C,^{11}B)$ = 56 Hz, C1), 128.6 (q, 3C, $^{1}J(^{13}C,^{11}B)$ = 65 Hz, CN), 115.4 (dq, 2C, $^{1}J(^{13}C,^{1}H)$ = 163 Hz, $^{2}J(^{13}C,^{11}B) \approx 4$ Hz, C2/6), 102.4 (dt, 1C, $^{1}J(^{13}C,^{1}H)$ = 166 Hz, $^{3}J(^{13}C,^{1}H)$ = 4.5 Hz, C4). $^{13}C\{^{13}B,^{1}H\}$ NMR (75.5 Hz, $(CD_3)_2CO$): $\delta = 163.6$ (dd, 2C, $^{1}J(^{19}F,^{13}C)$ = 248 Hz, $^{3}J(^{19}F,^{13}C)$ = 11.6 Hz, C3/5), 146.4 (t, 1C, $^{3}J(^{19}F,^{13}C)$ = 6.2 Hz, C1), 128.6 (s, 3C, CN), 115.4 (m, 2C, C2/6), 102.4 (t, 1C, $^{2}J(^{19}F,^{13}C)$ =25.6 Hz, C4). Elemental analysis: calculated (%) for $C_9H_3BF_2KN_3$, C 44.85, H 1.25, N 17.43; found, C 45.09, H 1.12, N 17.51. (−)-ESI-MS, m/z, $[(C_6F_2H_3)B(CN)_3]^-$: calculated 202.04 (100.0%), 201.04 (24.8%), 203.04 (9.7%); found 202.04 (100.0%), 201.04 (24.6%), 203.04 (9.5%). Crystals of K**B3d** suitable for an X-ray diffraction study were obtained from an acetone solution by slow evaporation of the solvent.

K[1-{(NC)$_3$B}-2,3,6-F$_3$-C$_6$H$_2$] (K**B4a**): <u>Method B</u> was employed for the preparation of

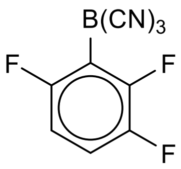

B4a

K**B4a**, using 1,2,3,4-tetrafluorobenzene (0.3 mL, 2.8 mmol), K_2**1** (225 mg, 1.35 mmol) and LiCl (85 mg, 2.01 mmol). The reaction mixture was stirred at room temperature for several minutes. Yield: 270 mg (1.04 mmol, 77%). ^{11}B NMR (160.5 MHz, $(CD_3)_2CO$): $\delta = -33.7$ ppm (dd, 1B, $^{3}J(^{19}F,^{11}B) \approx 8$ Hz (2×)). ^{1}H NMR (500.1 MHz, $(CD_3)_2CO$): δ = 7.21 (dddd, 1H, $^{3}J(^{1}H,^{1}H)$ = 9.1 Hz,

$^{3/4}J(^{19}F,^{1}H)$ = 9.9 Hz, 9.1 Hz, 5.0 Hz, H4), 6.85 ppm (m, 1H, H5). $^{1}H\{^{11}B\}$ NMR (500.1 MHz, $(CD_3)_2CO$): δ = 6.85 ppm (dddd, 1H, $^{3}J(^{1}H,^{1}H)$ = 9.1 Hz, $^{3/4/5}J(^{19}F,^{1}H)$ = 9.1 Hz, 8.8 Hz, 3.6 Hz, 2.2 Hz, H5) [other signal as stated for the ^{1}H NMR spectrum]. ^{19}F NMR (470.6 MHz, $(CD_3)_2CO$): δ = −106.8 (s, br, 1F, F6), −127.1 (s, br, 1F, F2), −145.6 ppm (m, 1F, F3). $^{13}C\{^{1}H\}$ NMR (125.8 MHz, $(CD_3)_2CO$): δ = 160.8 (ddd, 1C, $^{1}J(^{19}F,^{13}C)$ = 243 Hz, $^{3}J(^{19}F,^{13}C)$ = 10.6 Hz, $^{4}J(^{19}F,^{13}C)$ = 2.3 Hz, C6), 152.6 (ddd, 1C, $^{1}J(^{19}F,^{13}C)$ = 247 Hz, $^{2/3}J(^{19}F,^{13}C)$ = 13.4 Hz (2×), C2), 147.9 (dd, br, 1C, $^{1}J(^{19}F,^{13}C)$ = 242 Hz, $^{2}J(^{19}F,^{13}C)$ ≈ 15 Hz, C3), 127.5 (q, 3C, $^{1}J(^{13}C,^{11}B)$ = 67 Hz, CN), 117.2 (ddd, 1C, $^{2}J(^{19}F,^{13}C)$ = 19.6 Hz, $^{3}J(^{19}F,^{13}C)$ = 10.8 Hz, $^{4}J(^{19}F,^{13}C)$ = 1.6 Hz, C4), 116.4 (m, 1C, C1), 111.5 ppm (ddd, br, 1C, $^{3}J(^{19}F,^{13}C)$ = 28.2 Hz, 6.3 Hz, $^{4}J(^{19}F,^{13}C)$ = 4.0 Hz, C5). $^{13}C\{^{13}B,^{1}H\}$ NMR (75.5 Hz, $(CD_3)_2CO$): δ = 147.9 (ddd, 1C, $^{1}J(^{19}F,^{13}C)$ = 242 Hz, $^{2}J(^{19}F,^{13}C)$ = 15.5 Hz, $^{4}J(^{19}F,^{13}C)$ = 3.7 Hz, C3), 127.5 (dd, 3C, $^{4}J(^{19}F,^{13}C)$ = 1.4 Hz (2×), CN), 116.4 (ddd, $^{2}J(^{19}F,^{13}C)$ = 28.0 Hz, 21.6 Hz, $^{3}J(^{19}F,^{13}C)$ = 1.6 Hz, C1) [other signals as stated for the $^{13}C\{^{1}H\}$ NMR spectrum]. Elemental analysis: calculated (%) for $C_9H_2BF_3KN_3$, C 41.73, H 0.78, N 16.22; found, C 41.80, H 0.84, N 16.08. (−)-ESI-MS, m/z, $[(C_6F_3H_2)B(CN)_3]^-$: calculated 220.03 (100.0%), 219.03 (24.8%), 221.03 (9.7%); found 220.03 (100.0%), 219.03 (24.0%), 221.03 (9.5%). Crystals of K**B4a** suitable for an X-ray diffraction study were obtained from an acetone/water solution by slow evaporation of acetone.

K[1-{(NC)$_3$B}-2,3,5-F$_3$-C$_6$H$_2$] (K**B4b**): **1. Entry**: Method B was employed for the preparation of K**B4b**, using 1,2,3,5-tetrafluorobenzene (0.4 mL, 2.8 mmol), K_2**1** (200 mg, 1.20 mmol) and LiCl (80.0 mg, 1.89 mmol). The reaction mixture was stirred at room temperature for 2 hrs. Yield: 195 mg (0.753 mmol, 63%). **2. Entry**: Method B was employed for the preparation of K**B4b**, using 1,2,3,5-tetrafluorobenzene (ca. 0.4 mL, 3.7 mmol) and K_2**1** (300 mg, 1.80 mmol) but without addition of LiCl. The reaction mixture was stirred at room temperature for 3 days and then at 75 °C for 30 hrs. Yield: 240 mg (0.926 mmol, 51%). The product contained 8% of other isomers. ^{11}B NMR (160.5 MHz, $(CD_3)_2CO$): δ = −31.2 ppm (s, 1B). ^{1}H NMR (500.1 MHz, $(CD_3)_2CO$): δ = 7.10–7.04 (m, 1H, H4), 7.05–6.99 ppm (m, br, 1H, H6). ^{19}F NMR (470.6 MHz, $(CD_3)_2CO$): δ = −117.8 (m, 1F, F5), −136.7 (m, 1F, F3), −137.2 ppm (m, 1F, F2). $^{13}C\{^{1}H\}$ NMR (125.8 MHz, $(CD_3)_2CO$): δ = 158.3 (dm, 1C, $^{1}J(^{19}F,^{13}C)$ = 244 Hz, C5), 150.6 (dm, 1C, $^{1}J(^{19}F,^{13}C)$ = 248 Hz, C3), 149.7 (ddd, 1C, $^{1}J(^{19}F,^{13}C)$ = 239 Hz, $^{2}J(^{19}F,^{13}C)$ = 10.6 Hz, $^{4}J(^{19}F,^{13}C)$ = 3.4 Hz, C2), 131.0 (qd, 1C, $^{1}J(^{13}C,^{11}B)$ = 55 Hz, $^{2}J(^{19}F,^{13}C)$ ≈ 22 Hz, C1), 127.5 (q, 3C, $^{1}J(^{13}C,^{11}B)$ = 66 Hz, CN), 115.6 (ddd, 1C, $^{2}J(^{19}F,^{13}C)$ = 22.4 Hz, $^{3}J(^{19}F,^{13}C)$

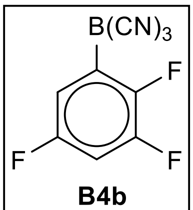

B4b

= 7.5 Hz, $^{4}J(^{19}F,^{13}C)$ = 3.5 Hz, C6), 105.0 ppm (ddd, 1C, $^{2}J(^{19}F,^{13}C)$ = 28.0 Hz, 21.4 Hz, C4). $^{13}C\{^{13}B,^{1}H\}$ NMR (75.5 Hz, $(CD_3)_2CO$): δ= 158.3 (ddd, $^{1}J(^{19}F,^{13}C)$ = 244 Hz, $^{3}J(^{19}F,^{13}C)$ = 9.4 Hz, $^{4}J(^{19}F,^{13}C)$ = 2.5 Hz, C5), 150.6 (m, 1C, C3), 149.7 (m, 1C, C2), 131.1 (dd, 1C, $^{2}J(^{19}F,^{13}C)$ = 21.7 Hz, $^{3}J(^{19}F,^{13}C)$ = 6.2 Hz, C1), 127.5 ppm (s, 3C, CN) [Signals for C4 and C6 as stated for the $^{13}C\{^{1}H\}$ NMR spectrum]. Elemental analysis: calculated (%) for $C_9H_2BF_3KN_3$, C 41.73, H 0.78, N 16.22; found, C 41.41, H 0.91, N 15.28. (−)-ESI-MS, m/z, $[(C_6F_3H_2)B(CN)_3]^-$: calculated 220.03 (100.0%), 219.03 (24.8%), 221.03 (9.7%); found 220.03 (100.0%), 219.03 (24.9%), 221.03 (9.4%). Crystals of K**B4b** suitable for an X-ray diffraction study were obtained from an acetone/water solution by slow evaporation of acetone.

K[1-{(NC)$_3$B}-2,4,5-F$_3$-C$_6$H$_2$] (K**B4c**) and **[Me$_3$NH][1-{(NC)$_3$B}-2,4,5-F$_3$-C$_6$H$_2$]** ([Me$_3$NH]-**B4c**): **1. Entry**: Method B was employed for the preparation of K**B4c**, using 1,2,4,5-tetrafluorobenzene (0.3 mL, 2.7 mmol), K_2**1** (200 mg, 1.20 mmol) and LiCl (80.0 mg, 1.89 mmol). The reaction mixture was stirred at room temperature for 30 min. Yield: 155 mg (0.598 mmol, 50%). **2. Entry**: [Me$_3$NH]**B4c**: Method B (modified) was employed for the preparation of [Me$_3$NH]**B4c**, using 1,2,4,5-tetrafluorobenzene (0.6 mL, 5.4 mmol), K_2**1** (310 mg, 1.86 mmol) and LiCl (120 mg, 2.83 mmol). The reaction mixture was stirred at room temperature for 2 hrs. The product was precipitated with slightly acidified aqueous solution of excess [Me$_3$NH]Cl, filtrated and dried in a vacuum. Yield: 260 mg (0.928 mmol, 50%). NMR spectroscopic data for K**B4c**: ^{11}B NMR (160.5 MHz, $(CD_3)_2CO$): δ = −31.2 ppm (s, 1B). ^{1}H NMR (500.1 MHz, $(CD_3)_2CO$): δ = 7.31 (m, br, 1H, H6), 7.05 ppm (ddd, 1H, $^{3/4}J(^{19}F,^{1}H)$ = 10.8 Hz, 8.6 Hz, 6.3 Hz, H3). $^{1}H\{^{11}B\}$ NMR (500.1 MHz, $(CD_3)_2CO$): δ = 7.31 (ddd, 1H, $^{3/4}J(^{19}F,^{1}H)$ = 11.0 Hz, 9.6 Hz, 5.8 Hz, H6) [signal for H3 as stated for the ^{1}H NMR spectrum]. ^{19}F NMR (470.6 MHz, $(CD_3)_2CO$): δ = −106.7 (s, br, 1F, F2), −137.8 (m, 1F, F4), −146.5 ppm (m, 1F, F5). $^{13}C\{^{1}H\}$ NMR (125.8 MHz, $(CD_3)_2CO$): δ= 160.9 (ddd, 1C, $^{1}J(^{19}F,^{13}C)$ = 243 Hz, $^{3}J(^{19}F,^{13}C)$ = 9.4 Hz, $^{4}J(^{19}F,^{13}C)$ = 2.2 Hz, C2), 150.1 (ddd, 1C, $^{1}J(^{19}F,^{13}C)$ = 248 Hz, $^{2}J(^{19}F,^{13}C)$ = 14.5 Hz, $^{3}J(^{19}F,^{13}C)$ = 13.1 Hz, C4), 147.2 (dm, br, 1C, $^{1}J(^{19}F,^{13}C)$ = 242 Hz, C5), 127.8 (qd, 3C, $^{1}J(^{13}C,^{11}B)$ = 66 Hz, $^{4}J(^{19}F,^{13}C) \approx$ 11 Hz, CN), 123.4 (qd, 1C, $^{1}J(^{13}C,^{11}B) \approx$ 56 Hz, $^{2}J(^{19}F,^{13}C)$ = 27 Hz, C1), 122.1 (dd, 1C, $^{2/3}J(^{19}F,^{13}C)$ = 18.1 Hz, 10.9 Hz, C6), 105.6 ppm (dd, 1C, $^{2/3}J(^{19}F,^{13}C)$ = 31.2 Hz, 20.3 Hz, C3). $^{13}C\{^{19}F\}$ NMR (125.8 MHz, $(CD_3)_2CO$): δ= 160.9 (dd, 1C, $^{2/3}J(^{13}C,^{1}H)$ = 13.8 Hz, 6.3 Hz, C2), 150.1 (dd, 1C, $^{2/3}J(^{13}C,^{1}H)$ = 9.3 Hz, 6.5 Hz, C4), 147.1 (s, br, 1C, C5), 127.1 (q, 3C, $^{1}J(^{13}C,^{11}B)$ = 66 Hz,

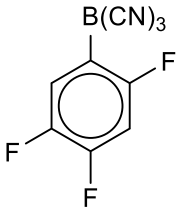

B4c

CN), 123.4 (q, 1C, $^1J(^{13}C,^{11}B) \approx 56$ Hz, C1), 122.1 (d, 1C, $^1J(^{13}C,^1H) = 164$ Hz, C6), 105.6 ppm (d, 1C, $^1J(^{13}C,^1H) = 166$ Hz, C3). Elemental analysis: calculated (%) for $C_{12}H_{12}BF_3N_4$, C 51.46, H 4.32, N 20.01; found, C 51.59, H 4.04, N 20.54. (−)-ESI-MS, m/z, $[(C_6F_3H_2)B(CN)_3]^-$: calculated 220.03 (100.0%), 219.03 (24.8%), 221.03 (9.7%); found 220.03 (100.0%), 219.03 (24.4%), 221.03 (9.4%). Crystals of [Me_3NH]**B4c** suitable for an X-ray diffraction study were obtained from an acetone/water solution by slow evaporation of acetone.

[Et_3NH]$_2$[1,4-{(NC)$_3$B}$_2$-2,5-F_2-C_6H_2] ([Et_3NH]$_2$**B4d**): $K_2B(CN)_3$ (K_2**1**, 300 mg, 1.80 mmol) and LiCl (115 mg, 2.71 mmol) was suspended in THF (7 mL). 1,2,4,5-tetrafluorobenzene (0.100 mL, 0.896 mmol) was added with a syringe in one portion. The reaction mixture was stirred at room temperature overnight. All solid materials that had formed were separated by filtration through a plug of Celite and washed with THF (2 × 5 mL). The Celite plug was washed with H_2O and upon addition of an aqueous solution with an excess of [Et_3NH]Cl to the aqueous layers (pH < 7) the main product precipitated. [Et_3NH]$_2$**B4d** was separated by filtration and dried in a vacuum. Yield: 185 mg (0.374 mmol, 42%, calculated for the fluoroarene). A second product crop was obtained from the combined THF layers: The solvent was removed first, the residue was dissolved in H_2O and the addition of an aqueous solution of [Et_3NH]Cl yielded 100 mg of a mixture of [Et_3NH]$_2$**B4d** and [Et_3NH]**B4c** (ratio according to ^{19}F NMR spectroscopy: 55:45). The NMR spectroscopic data for [Et_3NH]**B4c** match with those given above. ^{11}B NMR (160.5 MHz, $(CD_3)_2CO$): $\delta = -31.1$ ppm (s, 1B). ^1H NMR (500.1 MHz, $(CD_3)_2CO$): $\delta = 7.76$ (t, br, 2H, $^1J(^{14}N,^1H) = 49$ Hz, NH), 7.10 (dd, 2H, $^3J(^{19}F,^1H) = {}^4J(^{19}F,^1H) = 7.2$ Hz, H3/6), 3.41 (qd, 12H, $^3J(^1H,^1H) = 7.3$ Hz, 4.1 Hz, CH_2), 1.37 ppm (t, 18H, $^3J(^1H,^1H) = 7.3$ Hz, CH_3). ^{19}F NMR (470.6 MHz, $(CD_3)_2CO$): $\delta = -113.4$ ppm (t, 2F, $^3J(^{19}F,^1H) = 7.2$ Hz). $^{13}C\{^1H\}$ NMR (125.8 MHz, $(CD_3)_2CO$): $\delta = 162.2$ (dd, 2C, $^1J(^{19}F,^{13}C) = 241$ Hz, $^4J(^{19}F,^{13}C) = 3.5$ Hz, C2/5), 128.3 (q, 6C, $^1J(^{13}C,^{11}B) \approx 64$ Hz, CN), 127.5 (m, br, 2C, C1/4), 120.6 (m, 2C, C3/5), 48.0 (s, 6C, CH_2), 9.2 (s, 6C, CH_3). $^{13}C\{^{19}F\}$ NMR (125.8 MHz, $(CD_3)_2CO$): $\delta = 162.2$ (dd, 2C, $^{2/3}J(^{13}C,^1H) = 11.1$ Hz, 4.4 Hz, C2/5), 128.3 (q, 6C, $^1J(^{13}C,^{11}B) \approx 64$ Hz, CN), 127. 5 (m, br, 2C, C1), 120.6 (d, 2C, $^1J(^{13}C,^1H) = 161$ Hz, C3/5), 48.0 (t, 6C, $^1J(^{13}C,^1H) = 145$ Hz, CH_2), 9.2 (qt, 6C, $^1J(^{13}C,^1H) = 128$ Hz, $^2J(^{13}C,^1H) = 3.7$ Hz, CH_3). Elemental analysis: calculated (%) for $C_{24}H_{34}B_2F_2N_8$, C 58.33, H 6.93, N 22.67; found, C 57.95, H 6.80, N 21.14. (−)-ESI-MS, m/z, $[(C_6F_2H_2)\{B(CN)_3\}_2]^{2-}$: calculated 145.02 (100.0%), 144.53 (49.7%), 145.53 (13.0%); found 145.02 (100.0%), 144.53 (48.9%), 145.53

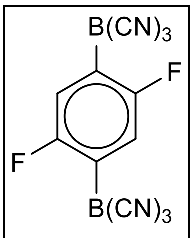

B4d

(12.1%). Crystals of $[Et_3NH]_2$**B4d** suitable for an X-ray diffraction study were obtained from an acetone/water solution by slow evaporation of acetone.

K[1-{(NC)$_3$B}-2,3,5,6-F$_4$-C$_6$H] (K**B5**): **1. Entry**: Method B was employed for the preparation of K**B5**, using pentafluorobenzene (0.3 mL, 2.7 mmol), K_2**1** (200 mg, 1.20 mmol) and LiCl (80.0 mg, 1.89 mmol). The arene was added at −78 °C, warmed up to room temperature, and the reaction mixture was stirred for 10 minutes. Yield: 205 mg (0.740 mmol, 62%). The product contained ca. 6% of another isomer. **2. Entry**: Method B was employed for the preparation of K**B5**, using pentafluorobenzene (0.5 mL, 4.5 mmol) and K_2**1** (350 mg, 2.08 mmol) without addition of LiCl. The arene was added at −78 °C, warmed up to room temperature, and the reaction mixture was stirred for 2 days. Yield: 225 mg (0.812 mmol, 39%). The product contained ca. 20% of other isomers and $[BH(CN)_3]^-$. ^{11}B NMR (160.5 MHz, $(CD_3)_2CO$): $\delta = -33.7$ ppm (tt, 1B, $^3J(^{19}F,^{11}B) = 7.6$ Hz, $^4J(^{19}F,^{11}B) = 3.1$ Hz). ^{1}H NMR (500.1 MHz, $(CD_3)_2CO$): $\delta = 7.33$ (tt, 1H, $^3J(^{19}F,^{1}H) = 10.1$ Hz, $^4J(^{19}F,^{1}H) = 7.5$ Hz). ^{19}F NMR (470.6 MHz, $(CD_3)_2CO$): $\delta = -132.7$ (m, 2F, F2/6), –141.8 ppm (m, 2F, F3/5). $^{13}C\{^1H\}$ NMR (125.8 MHz, $(CD_3)_2CO$): $\delta = 148.3$ (dddd, 2C, $^1J(^{19}F,^{13}C) = 244$ Hz, $^{3/4}J(^{19}F,^{13}C) \approx 13$ Hz, 11 Hz, $^5J(^{19}F,^{13}C) \approx 4$ Hz, C2/6), 146.5 (dm, 2C, $^1J(^{19}F,^{13}C) = 247$ Hz, C3/5), 127.0 (q, 3C, $^1J(^{13}C,^{11}B) = 67$ Hz, CN), 118.3 (qt, 1C, $^1J(^{13}C,^{11}B) = 56$ Hz, $^2J(^{19}F,^{13}C) = 24$ Hz, C1), 106.1 ppm (tt, 1C, $^2J(^{19}F,^{13}C) = 23.2$ Hz, $^3J(^{19}F,^{13}C) = 1.4$ Hz, C4). $^{13}C\{^{19}F\}$ NMR (125.8 MHz, $(CD_3)_2CO$): $\delta = 148.3$ (d, 2C, $^3J(^{13}C,^{1}H) = 9.0$ Hz, C2/6), 146.5 (m, 2C, C3/5), 127.0 (q, 3C, $^1J(^{13}C,^{11}B) = 67$ Hz, CN), 118.3 (q, 1C, $^1J(^{13}C,^{11}B) = 56$ Hz, C1), 106.1 ppm (d, 1C, $^1J(^{13}C,^{1}H) = 169$ Hz, C4). Elemental analysis: calculated (%) for $C_9HBF_4KN_3$, C 39.02, H 0.36, N 15.17; found, C 40.02, H 0.42, N 14.12. (−)-ESI-MS, m/z, $[(C_6F_4H)B(CN)_3]^-$: calculated 238.02 (100.0%), 237.02 (24.8%), 239.02 (9.7%); found 238.02 (100.0%), 237.02 (24.1%), 239.02 (9.3%).

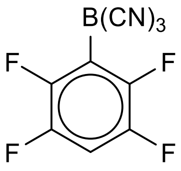

B5

K[1-{(NC)$_3$B}-C$_6$F$_5$] (K**B6a**): The preparation of K**B6a** was performed as described in the literature and the NMR spectroscopic data are consistent to the published ones.[10] (−)-ESI-MS, m/z, $[(C_6F_5)B(CN)_3]^-$: calculated 256.01 (100.0%), 255.01 (24.8%), 257.01 (9.7%), found 256.01 (100.0%), 255.01 (24.7%), 257.01 (9.5%).

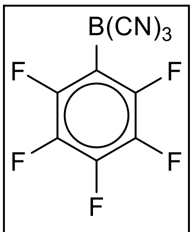

B6a

$K_2[1,4\text{-}\{(NC)_3B\}_2\text{-}C_6F_4]$ (K_2B6b): Method A was employed for the preparation of K_2**B6b** using hexafluorobenzene (2.30 mL, 19.9 mmol), K_2**1** (7.20 g, 43.1 mmol) and THF (50 mL). The reaction mixture was heated to reflux for 20 hrs. The Celite plug was extracted with THF (2 × 50 mL) and CH_3CN (200 mL) due to the low solubility of K_2**B6b** in THF. Yield: 5.95 g (14.7 mmol, 74%, calculated for C_6F_6). The following analytical data were obtained for $[Et_4N]_2$[**B6b**] which was precipitated from an aqueous solution of K_2**B6b** with aqueous $[Et_4N]OH$, filtered off and dried in a vacuum. ^{11}B NMR (160.5 MHz, $(CD_3)_2CO$): $\delta = -34.7$ ppm (s, 2B). ^{19}F NMR (470.6 MHz, $(CD_3)_2CO$): $\delta = -133.8$ ppm (s, 4F). $^{13}C\{^1H\}$ NMR (125.8 MHz, $(CD_3)_2CO$): $\delta = 148.2$ (dm, 4C, $^1J(^{19}F,^{13}C) \approx 250$ Hz, C2/3/5/6), 127.4 (q, 6C, $^1J(^{13}C,^{11}B) = 67$ Hz, CN), 116.7 ppm (m, 2C, C1/C4). $^{13}C\{^{13}B,^1H\}$ NMR (75.5 Hz, $(CD_3)_2CO$): $\delta = 148.2$ (dm, 4C, $^1J(^{19}F,^{13}C) = 247$ Hz, C2/3/5/6), 127.4 (s, 6C, CN), 116.6 ppm (m, 2C, C1/C4). Elemental analysis: calculated (%) for $C_{28}H_{40}B_2F_4N_8$, C 57.36, H 6.88, N 19.11; found, C 57.36, H 6.97, N 18.35. (−)-ESI-MS, m/z, $[(C_6F_4)\{B(CN)_3\}_2]^{2-}$: calculated 163.02 (100.0%), 162.52 (49.7%), 163.52 (13.0%); found 163.02 (100.0%), 162.52 (47.3%), 163.52 (11.9%). Crystals of $[Et_4N]_2$[**B6b**] suitable for an X-ray diffraction study were obtained from an acetone solution by slow evaporation of the solvent.

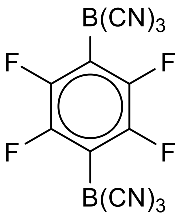

B6b

$K[4\text{-}\{(NC)_3B\}\text{-}C_5F_4N]$ (K**Py1**): Method A was employed for the preparation of K**Py1** using pentafluoropyridine (ca. 0.8 mL, 7.3 mmol), K_2**1** (400 mg, 2.39 mmol) and THF (15 mL). The pyridine was added at −40 °C and the reaction mixture was stirred at room temperature for 12 hrs. Yield: 575 mg (1.94 mmol, 81%; contained ca. 15% of other tricyano(fluoropyridinyl)borates). ^{11}B NMR (160.5 MHz, $(CD_3)_2CO$): $\delta = -33.9$ ppm (tt, 1B, $^3J(^{19}F,^{11}B) = 6.2$ Hz, $^4J(^{19}F,^{11}B) \approx 2.5$ Hz). ^{19}F NMR (470.6 MHz, $(CD_3)_2CO$): $\delta = -96.0$ (m, 2F, F2/6), −134.7 ppm (m, 2F, F3/5). $^{13}C\{^1H\}$ NMR (125.8 MHz, $(CD_3)_2CO$): $\delta = 145.2$–142.8 (m, 4C, C2/3/5/6), 133.5 (m, 1C, C4), 125.8 ppm (q, 3C, $^1J(^{13}C,^{11}B) \approx 68$ Hz, CN). $^{13}C\{^{19}F\}$ NMR (125.8 MHz, $(CD_3)_2CO$): $\delta = 144.1$ (s, 2C, C3/5), 144.0 (s, 2C, C2/6), 133.7 (q, 1C, $^1J(^{13}C,^{11}B) = 54$ Hz, C4), 125.9 ppm (q, 3C, $^1J(^{13}C,^{11}B) = 68$ Hz, CN). $^{13}C\{^{13}B,^1H\}$ NMR (75.5 Hz, $(CD_3)_2CO$): $\delta = 145.6$–141.7 (m, 4C, C2/3/5/6), 132.5 (tt, 1C, $^2J(^{19}F,^{13}C) = 21.7$ Hz, $^3J(^{19}F,^{13}C) = 1.1$ Hz, C4), 125.6 ppm (t, 3C, $^4J(^{19}F,^{13}C) = 1.1$ Hz, CN). (−)-ESI-MS, m/z, $[(NC_5F_4)B(CN)_3]^-$: calculated 239.02 (100.0%), 238.02 (24.8%), 240.02 (8.7%); found 239.02 (100.0%), 238.02 (24.4%), 240.02

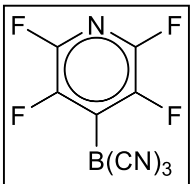

Py1

(8.6%). Crystals of K**Py1** suitable for an X-ray diffraction study were obtained by slow diffusion of dichloromethane into an acetone solution.

K_2[2,4-{(NC)$_3$B}$_2$-C_5F_3N] (K_2**Py2**): Method A was employed for the preparation of K_2**Py2**

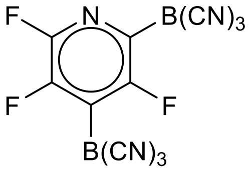

Py2

using pentafluoropyridine (0.160 mL, 1.46 mmol), K_2**1** (500 mg, 2.99 mmol) and THF (15 mL). The pyridine was added at −60 °C and the reaction mixture was stirred for 4 days. Addition of CH_2Cl_2 to the combined THF layers resulted in two fractions. The first fraction was pure K_3**Py3** (85.0 mg, 0.171 mmol, 12%; for the analytical data see the following experiment). Yield (2. fraction, only): 335 mg (0.866 mmol, 59%; calculated for C_5F_5N; contained 15% of K_3**Py3** and 10% of another tricyano(fluoropyridinyl)borate). ^{11}B NMR (160.5 MHz, $(CD_3)_2CO$): δ = −30.9 (s, 1B, B2), −33.9 ppm (s, 1B, B4). ^{19}F NMR (470.6 MHz, $(CD_3)_2CO$): δ = −94.7 (dd, 1F, $^{3/5}J(^{19}F,^{19}F)$ = 30.0 Hz, 32.3 Hz, F6), −108.3 (m, 1F, F3), −132.4 ppm (m, 1F, F5). $^{13}C\{^1H\}$ NMR (125.8 MHz, $(CD_3)_2CO$): δ = 161.9 (d, 1C, $^1J(^{19}F,^{13}C) \approx$ 253 Hz, C3), 147.9 (dm, 1C, $^1J(^{19}F,^{13}C) \approx$ 230 Hz, C6), 146.3 (ddd, 1C, $^1J(^{19}F,^{13}C)$ = 259 Hz, $^{2/3}J(^{19}F,^{13}C)$ = 32.0 Hz, 8.6 Hz, C5), 142.8 (m, br, 1C, C2), 130.4–125.7 ppm (m, 7C, C4/CN). (−)-ESI-MS, m/z, $[(NC_5F_3)\{B(CN)_3\}_2]^{2-}$: calculated 154.52 (100.0%), 154.02 (49.7%), 155.02 (11.9%); found 154.52 (100.0%), 154.02 (47.9%), 155.02 (11.2%). Crystals of K_2**Py2**·$OC(CH_3)_2$ suitable for an X-ray diffraction study were obtained from an acetone solution by slow evaporation of the solvent.

K_3[2,4,6-{(NC)$_3$B}$_3$-C_5F_2N] (K_3**Py3**): Method A was employed for the preparation of K_3**Py3**

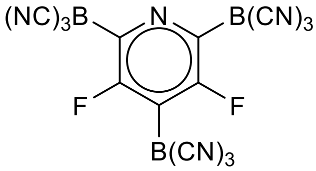

Py3

using pentafluoropyridine (0.108 mL, 0.988 mmol), K_2**1** (500 mg, 2.99 mmol) and THF (10 mL). The pyridine was added at −78 °C and the reaction mixture was stirred at room temperature for four days. Upon addition of CH_2Cl_2 to the combined THF layers two fractions were obtained. The first fraction was pure K_3**Py3** and the second fraction was a mixture of K_3**Py3** and K_2**Py2** (4:6; 150 mg, 0.340 mmol, 34%). Yield (1. fraction, only): 150 mg (0.302 mmol, 31%, calculated for C_5F_5N). ^{11}B NMR (160.5 MHz, $(CD_3)_2CO$): δ = −30.0 (s, br, 2B, B2/6), −33.9 ppm (s, 1B, B4). ^{19}F NMR (470.6 MHz, $(CD_3)_2CO$): δ = −107.5 ppm (s, 2F). $^{13}C\{^1H\}$ NMR (125.8 MHz, $(CD_3)_2CO$): δ = 164.7 (dd, 2C, $^1J(^{19}F,^{13}C)$ = 258 Hz, $^3J(^{19}F,^{13}C)$ = 5.7 Hz, C3/5), 146.4 (m, 2C, C2/6), 128.3 (m, 9C, CN), 118.4 ppm (m, 1C, C4). (−)-ESI-MS, m/z, $[(NC_5F_2)\{B(CN)_3\}_3]^{3-}$: calculated 126.35 (100.0%), 126.02 (66.9%), 125.69 (16.6%), 126.69 (13.6%); found 126.35 (100.0%), 126.02 (69.6%), 125.69 (16.7%),

126.69 (13.7%). Crystals of K_3**Py3**·3THF·1.04H_2O suitable for an X-ray diffraction study were obtained by slow diffusion of hexane in a THF solution.

K[4-{(NC)$_3$B}-$C_{12}F_9$] (K**BP1**): <u>Method A</u> was employed for the preparation of K**BP1** using decafluorobiphenyl (750 mg, 2.24 mmol), K_2**1** (300 mg, 1.80 mmol) and THF (15 mL). The reaction mixture was stirred at room temperature for 2 hrs. Upon addition of CH_2Cl_2 to the combined THF layers two fractions were obtained. The first fraction contained impurities (mainly K_2**BP2**, see next example) and was neglected. Yield (2. fraction, only): 390 mg (0.880 mmol, 49%). ^{11}B NMR (160.5 MHz, $(CD_3)_2CO$): δ = −33.7 ppm (s, 1B). ^{19}F NMR (470.6 MHz, $(CD_3)_2CO$): δ = −131.4 (m, 2F, F3/5), −139.8 (m, 2F, F2'/6'), −141.6 (m, 2F, F2/6), −153.2 (tt, 1F, $^3J(^{19}F, ^{19}F)$ = 20.5 Hz, $^4J(^{19}F, ^{19}F)$ = 3.0 Hz, F4'), −163.3 ppm (m, 2F, F3'/5'). $^{13}C\{^1H\}$ NMR (125.8 MHz, $(CD_3)_2CO$): δ = 148.6 (ddd, 2C, $^1J(^{19}F,^{13}C)$ = 245 Hz, $^{2/3}J(^{19}F,^{13}C)$ = 12 Hz, 4 Hz, C3/5), 145.4 (dm, 2C, $^1J(^{19}F,^{13}C)$ = 250 Hz, C2'/6'), 144.6 (dm, br, 2C, $^1J(^{19}F,^{13}C)$ = 251 Hz, C2/6), 143.3 (tt, 1C, $^1J(^{19}F,^{13}C)$ = 254 Hz, $^2J(^{19}F,^{13}C)$ = 13.5 Hz, $^3J(^{19}F,^{13}C)$ = 5.2 Hz, C4'), 138.9 (dm, 2C, $^1J(^{19}F,^{13}C)$ = 250 Hz, C3'/C5'), 126.7 (q, 3C, $^1J(^{13}C,^{11}B)$ = 68 Hz, CN), 121.1 (qt, 1C, $^1J(^{13}C,^{11}B) \approx$ 55 Hz, $^2J(^{19}F,^{13}C) \approx$ 25 Hz, C4), 105.8 (t, 1C, $^2J(^{19}F,^{13}C)$ = 18 Hz, C1'), 103.6 ppm (t, 1C, $^2J(^{19}F,^{13}C)$ = 19 Hz, C1). $^{13}C\{^{19}F\}$ NMR (125.8 MHz, $(CD_3)_2CO$): δ = 148.6 (s, 2C, C3/5), 145.4 (s, 2C, C2'/6'), 144.6 (s, br, 2C, C2/6), 143.3 (s, 1C, C4'), 138.9 (s, 2C, C3'/C5'), 126.7 (q, 3C, $^1J(^{13}C,^{11}B)$ = 68 Hz, CN), 121.1 (q, 1C, $^1J(^{13}C,^{11}B)$ = 55 Hz, C4), 105.8 (s, 1C, C1'), 103.6 ppm (s, 1C, C1). Elemental analysis: calculated (%) for $C_{15}BF_9KN_3$, C 40.66, N 9.48; found, C 40.81, N 9.81. (−)-ESI-MS, m/z, $[(C_{12}F_9)B(CN)_3]^-$: calculated 404.00 (100.0%), 403.01 (24.8%), 405.01 (16.2%); found 404.00 (100.0%), 403.01 (25.0%), 405.01 (16.3%).

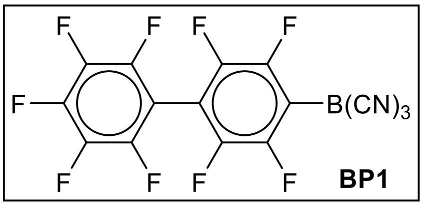

K_2[4,4'-{(NC)$_3$B}$_2$-$C_{12}F_8$] (K_2**BP2**): <u>Method A</u> was employed for the preparation of K_2**BP2** using decafluorobiphenyl (450 mg, 1.35 mmol), K_2**1** (500 mg, 2.99 mmol) and THF (10 mL). The reaction mixture was stirred at 50 °C for 1 hr. Yield: 675 mg (1.22 mmol, 90%, calculated for $C_{10}F_{10}$). ^{11}B NMR (160.5 MHz, $(CD_3)_2CO$): δ = −33.7 ppm (s, 2B). ^{19}F NMR (470.6 MHz, $(CD_3)_2CO$): δ = −131.7 (s, br, 4F, F3/5), −141.7 ppm (m, 4F, F2/6). $^{13}C\{^1H\}$ NMR (125.8 MHz, $(CD_3)_2CO$): δ = 148.5 (dm, 4C, $^1J(^{19}F,^{13}C) \approx$ 245 Hz, C3/5), 144.6 (dd, 4C, $^1J(^{19}F,^{13}C) \approx$ 250 Hz, $^2J(^{19}F,^{13}C)$

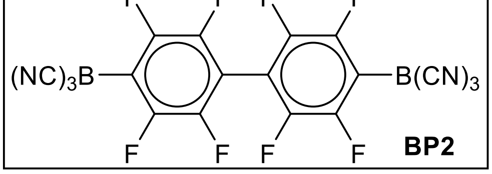

≈ 18 Hz, C2/6), 126.7 (q, 6C, $^{1}J(^{13}C,^{11}B)$ = 67 Hz, CN), 120.3 (m, 2C, C4), 107.1 ppm (m, 2C, C1). $^{13}C\{^{19}F\}$ NMR (125.8 MHz, $(CD_3)_2CO$): δ = 148.5 (s, 4C, C3/5), 144.5 (s, 4C, C2/6), 126.7 (q, 6C, $^{1}J(^{13}C,^{11}B)$ ≈ 66 Hz, CN), 120.3 (q, br, 2C, $^{1}J(^{13}C,^{11}B)$ ≈ 53 Hz, C4), 107.1 ppm (s, 2C, C1). $^{13}C\{^{13}B,^{1}H\}$ NMR (75.5 Hz, $(CD_3)_2CO$): δ = 148.5 (dm, 4C, $^{1}J(^{19}F,^{13}C)$ ≈ 246 Hz, C3/5), 144.6 (dd, 4C, $^{1}J(^{19}F,^{13}C)$ ≈ 250 Hz, $^{2}J(^{19}F,^{13}C)$ ≈ 18 Hz, C2/6), 126.7 (t, 6C, $^{4}J(^{19}F,^{13}C)$ = 1.3 Hz, CN), 120.3 (t, 2C, $^{2}J(^{19}F,^{13}C)$ = 23.8 Hz, C4), 107.1 ppm (m, 2C, C1). Elemental analysis: calculated (%) for $C_{18}B_2F_8K_2N_6$, C 39.16, N 15.22; found, C 39.05, N 14.62. (−)-ESI-MS, m/z, $[(C_{12}F_8)\{B(CN)_3\}_2]^{2-}$: calculated 237.01 (100.0%), 236.51 (49.7%), 237.51 (19.5%), 237.02 (9.7%); found 237.01 (100.0%), 236.51 (44.9%), 237.51 (17.2%). Crystals of K_2**BP2**·THF suitable for an X-ray diffraction study were obtained by slow diffusion of hexane into a THF solution.

K[2-{(NC)$_3$B}-C$_{10}$F$_7$] (K**N1**): Method A was employed for the preparation of K**N1** using octafluoronaphthalene (650 mg, 2.39 mmol) and K_2**1** (200 mg, 1.20 mmol). The reaction mixture was stirred at room temperature for 3 days. The crude product was washed with CH_2Cl_2 (100 mL) and then dissolved in THF (10 mL) again. Addition of CH_2Cl_2 resulted in solid K**N1**. Yield: 250 mg (0.656 mmol, 55%).

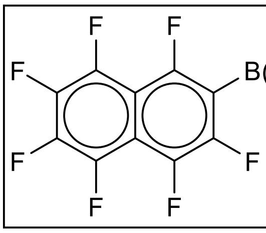

N1

^{11}B NMR (160.5 MHz, $(CD_3)_2CO$): δ = −33.6 ppm (dd, 1B, $^{2}J(^{19}F,^{11}B)$ ≈ 8 Hz (2×)). ^{19}F NMR (470.6 MHz, $(CD_3)_2CO$): δ = −107.3 (d, 1F, $^{4}J(^{19}F,^{19}F)$ = 74 Hz, F1), −127.3 (s, 1F, F3), −146.3 (dddm, 1F, $^{4}J(^{19}F,^{19}F)$ = 74 Hz, $^{3}J(^{19}F,^{19}F)$ ≈ $^{5}J(^{19}F,^{19}F)$ ≈ 17 Hz, F8), −149.4 (dddd, 1F, $^{4}J(^{19}F,^{19}F)$ = 56 Hz, $^{3}J(^{19}F,^{19}F)$ ≈ $^{5}J(^{19}F,^{19}F)$ ≈ 16 Hz, $J(^{19}F,^{19}F)$ = 5 Hz, F5), −153.3 (dddm, 1F, $^{4}J(^{19}F,^{19}F)$ = 56 Hz, $^{3}J(^{19}F,^{19}F)$ ≈ $^{5}J(^{19}F,^{19}F)$ ≈ 18 Hz, F4), −157.3 (ddm, 1F, $^{3}J(^{19}F,^{19}F)$ ≈ $^{3}J(^{19}F,^{19}F)$ ≈ 18 Hz, F6), −159.6 ppm (ddm, 1F, $^{3}J(^{19}F,^{19}F)$ ≈ $^{3}J(^{19}F,^{19}F)$ ≈ 18 Hz, F7). $^{13}C\{^{1}H\}$ NMR (125.8 MHz, $(CD_3)_2CO$): δ = 154.4 (d, 1C, $^{1}J(^{19}F,^{13}C)$ = 255 Hz, C3), 150.5 (ddd, 1C, $^{1}J(^{19}F,^{13}C)$ = 248 Hz, $J(^{19}F,^{13}C)$ ≈ 12 Hz (2×)), 142.2 (dm, 1C, $^{1}J(^{19}F,^{13}C)$ ≈ 255 Hz), 141.7 (dm, 1C, $^{1}J(^{19}F,^{13}C)$ = 255 Hz), 140.9 (dm, 1C, $^{1}J(^{19}F,^{13}C)$ = 252 Hz), 140.2 (ddd, br, 1C, $^{1}J(^{19}F,^{13}C)$ = 252 Hz, $J(^{19}F,^{13}C)$ ≈ 15 Hz (2×)), 139.1 (ddd, br, 1C, $^{1}J(^{19}F,^{13}C)$ = 250 Hz, $J(^{19}F,^{13}C)$ ≈ 15 Hz (2×)), 127.0 (q, 3C, $^{1}J(^{13}C,^{11}B)$ ≈ 67 Hz, CN), 116.8 (qd, 1C, $^{1}J(^{13}C,^{11}B)$ ≈ 56 Hz, $^{2}J(^{19}F,^{13}C)$ ≈ 28 Hz, C2), 112.2 (dd, 1C, $J(^{19}F,^{13}C)$ ≈ 12 Hz (2×), C4a), 108.9 ppm (dd, 1C, $J(^{19}F,^{13}C)$ ≈ 19 Hz, 12 Hz, C8a) [A definite assignment even with the aid of calculated chemical shifts was not possible]. $^{13}C\{^{19}F\}$ NMR (125.8 MHz, $(CD_3)_2CO$): δ = 154.4 (s, 1C), 150.5 (s, 1C), 142.2 (s, 1C), 141.7 (s, 1C), 140.2 (s, 1C), 140.9 (s, br, 1C), 139.1 (s, 1C), 127.0 (q, 3C, $^{1}J(^{13}C,^{11}B)$ ≈ 67 Hz, CN), 116.8 (q, 1C, $^{1}J(^{13}C,^{11}B)$ ≈ 56 Hz, C2),

112.2 (s, 1C, C4a), 108.9 ppm (s, 1C, C8a) [A definite assignment even with the aid of calculated chemical shifts was not possible]. Elemental analysis: calculated (%) for $C_{13}BF_7KN_3$, C 40.98, N 11.03; found, C 40.81, N 11.01. (−)-ESI-MS, m/z, $[(C_{10}F_7)B(CN)_3]^-$: calculated 342.01 (100.0%), 341.01 (24.8%), 343.01 (14.1%); found 342.01 (100.0%), 341.01 (23.2%), 343.01 (13.3%).

$K_2[2,6\text{-}\{(NC)_3B\}_2\text{-}C_{10}F_6]$ (K_2**N2**): Modified Method A was employed for the preparation of K_2**N2** using octafluoro-naphthalene (163 mg, 0.599 mmol), K_2**1** (200 mg, 1.20 mmol) and THF (5 mL).

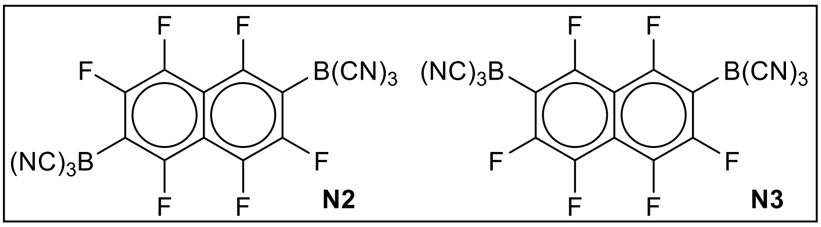

The reaction mixture was stirred at 60 °C for 16 hrs. All solids that had formed were separated by filtration through a plug of Celite and washed with cold THF (3 × 1.5 mL). Then, the plug was washed with H_2O (10 mL), and after reducing the volume and cooling to 0 °C small colourless needles had formed. These were decanted and dried in a vacuum. Yield: 70.0 mg (0.143 mmol, 24%; calculated for $C_{10}F_8$). The combined THF layers contained additional product which could not be separated from other isomers formed. The total ratio of the borates formed was 0.4:1.0:0.7 (K**N1**:K_2**N2**:K_2**N3**; **N3** = $[2,7\text{-}\{(NC)_3B\}_2\text{-}C_{10}F_6]^{2-}$). NMR spectroscopic data for K_2**N2**: ^{11}B NMR (160.5 MHz, $(CD_3)_2CO$): δ = −33.6 ppm (dd, 1B, $^2J(^{19}F,^{11}B) \approx 8$ Hz (2×)). ^{19}F NMR (470.6 MHz, $(CD_3)_2CO$): δ = −108.8 (m, 2F, F1/5), −130.1 (s, 2F, F3/7), −152.1 ppm (m, 2F, F4/8). $^{13}C\{^{19}F\}$ NMR (125.8 MHz, $(CD_3)_2CO$): δ = 154.2 (s, 2C, C1/5), 149.7 (s, 2C, C3/7), 140.9 (s, 2C, C4/8), 127.3 (q, 6C, $^1J(^{13}C,^{11}B)$ = 67 Hz, CN), 116.7 (q, 2C, $^1J(^{13}C,^{11}B)$ = 55 Hz, C2/6), 112.9 ppm (s, 2C, C4a/8a). Elemental analysis (the salt used contained one equivalent of H_2O): calculated (%) for $C_{16}H_2B_2F_6K_2N_6O$, C 37.83, H 0.40, N 16.54; found, C 37.65, H 0.25, N 15.38. (−)-ESI-MS, m/z, $[(C_{10}F_6)\{B(CN)_3\}_2]^{2-}$: calculated 206.01 (100.0%), 205.52 (49.7%), 206.52 (17.3%); found 206.01 (100.0%), 205.52 (48.9%), 206.51 (16.0%). Crystals of K_2**N2**·2EtOAc suitable for an X-ray diffraction study were obtained from an ethyl acetate solution by slow evaporation of the solvent. NMR spectroscopic data for K_2**N3**: ^{11}B NMR (160.5 MHz, $(CD_3)_2CO$): δ = ^{11}B NMR (160.5 MHz, $(CD_3)_2CO$): [the signal is covered by the signal of anion **N2**]. ^{19}F NMR (470.6 MHz, $(CD_3)_2CO$): δ = −104.6 (m, 2F, F1/8), −127.6 (m, 2F, F3/6), −154.5 ppm (m, 2F, F4/5). [In the ^{13}C NMR spectra the signals of anion **N3** were not observed.]

K[1-F_3C-4-{(NC)$_3$B}-C_6F_4] (K**B7**): Method A was employed for the preparation of K**B7** using octafluorotoluene (ca. 0.3 mL, 2.1 mmol) and K_2**1** (150 mg, 0.898 mmol). The reaction mixture was stirred at room temperature for 3 hrs. Yield: 210 mg (0.609 mmol, 68%). ^{11}B NMR (160.5 MHz, $(CD_3)_2CO$): $\delta = -33.8$ ppm (tt, 1B, $^3J(^{19}F,^{11}B) = 7.3$ Hz, $^4J(^{19}F,^{11}B) = 2.8$ Hz). ^{19}F NMR (470.6 MHz, $(CD_3)_2CO$): $\delta = -57.2$ (tt, 3F, $^4J(^{19}F,^{19}F) = 22$ Hz, $^5J(^{19}F,^{19}F) = 0.6$ Hz, CF_3), −130.4 (m, 2F, C3/5), −144.3 ppm (m, 2F, C2/6). $^{13}C\{^1H\}$ NMR (125.8 MHz, $(CD_3)_2CO$): $\delta = 148.8$ (dm, 2C, $^1J(^{19}F,^{13}C) = 246$ Hz, C3/5), 144.5 (dm, 2C, $^1J(^{19}F,^{13}C) = 257$ Hz, C2/6), 126.4 (q, 3C, $^1J(^{13}C,^{11}B) = 68$ Hz, CN), 123.6 (qm, 1C, $^1J(^{13}C,^{11}B) = 55$ Hz, C4), 122.1 (qm, 1C, $^1J(^{19}F,^{13}C) = 273$ Hz, CF_3), 108.8 ppm (qt, 1C, $^2J(^{19}F,^{13}C) = 34$ Hz, $^2J(^{19}F,^{13}C) = 13$ Hz, C1). $^{13}C\{^{13}B,^1H\}$ NMR (75.5 Hz, $(CD_3)_2CO$): $\delta = 148.8$ (dm, 2C, $^1J(^{19}F,^{13}C) = 246$ Hz, C3/5), 144.5 (dm, 2C $^1J(^{19}F,^{13}C) = 257$ Hz, C2/6), 126.3 (t, 3C, $^4J(^{19}F,^{13}C) = 1.4$ Hz, CN), 123.5 (t, 1C, $^2J(^{19}F,^{13}C) \approx 24$ Hz, C4), 122.1 (qtt, 1C, $^1J(^{19}F,^{13}C) = 273$ Hz, $^3J(^{19}F,^{13}C) \approx 3.5$ Hz, $^4J(^{19}F,^{13}C) \approx 1.5$ Hz, CF_3), 108.8 ppm (qt, 1C, $^2J(^{19}F,^{13}C) = 34$ Hz, $^2J(^{19}F,^{13}C) = 13$ Hz, C1). Elemental analysis: calculated (%) for $C_{10}BF_7KN_3$, C 34.81, N 12.18; found, C 34.41, N 12.41. (−)-ESI-MS, m/z, $[(CF_3C_6F_4)\{B(CN)_3\}]^-$: calculated 306.01 (100.0%), 305.01 (24.8%), 307.01 (10.8%); found 306.01 (100.0%), 305.01 (23.4%), 307.01 (10.5%). Crystals of K**B7**·THF suitable for an X-ray diffraction study were obtained from a THF solution by slow evaporation of the solvent.

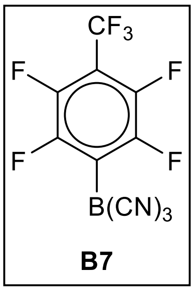

K[1-H_3C-4-{(NC)$_3$B}-C_6F_4] (K**B8a**): Method A was employed for the preparation of K**B8a** using 2,3,4,5,6-pentafluorotoluene (ca. 0.18 mL, 1.4 mmol) and K_2**1** (200 mg, 1.20 mmol). The reaction mixture was stirred at 90 °C for 3 days. Yield: not determined. The product contained ca. 10% of K[1-H_3C-4-{(NC)$_3$B}-C_6F_4] (K**B8b**). NMR spectroscopic data for K**B8a**: ^{11}B NMR (160.5 MHz, $(CD_3)_2CO$): $\delta = -33.8$ ppm (tt, 1B, $^3J(^{19}F,^{11}B) = 7.5$ Hz, $^4J(^{19}F,^{11}B) = 3.2$ Hz). ^{19}F NMR (470.6 MHz, $(CD_3)_2CO$): $\delta = -134.1$ (m, 2F, F3/5), −146.7 ppm (dd, 2F, $^{2/3}J(^{19}F,^{19}F) = 22$ Hz, 14 Hz, F2/6). ^{1}H NMR (500.1 MHz, $(CD_3)_2CO$): $\delta = 2.23$ ppm (t, 3H, $^4J(^{19}F,^1H) = 2.1$ Hz). $^{13}C\{^1H\}$ NMR (125.8 MHz, $(CD_3)_2CO$): $\delta = 148.1$ (dddd, 2C, $^1J(^{19}F,^{13}C) = 243$ Hz, $^{2/3}J(^{19}F,^{13}C) \approx 14$ Hz, 12 Hz, $^4J(^{19}F,^{13}C) \approx 4$ Hz, C3/5), 145.6 (dm, br, 2C, $^1J(^{19}F,^{13}C) = 243$ Hz, C2/6), 127.3 (q, 3C, $^1J(^{13}C,^{11}B) = 67$ Hz, CN), 116.0 (t, 1C, $^2J(^{19}F,^{13}C) = 19.4$ Hz, C1), 115.3–113.6 (m, 1C, C4), 7.3 ppm (tt, 1C, $^3J(^{19}F,^{13}C) = 3.5$ Hz,

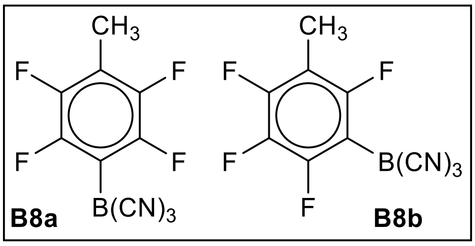

$J(^{19}F,^{13}C)$ = 2.2 Hz, CH_3). $^{13}C\{^{19}F\}$ NMR (125.8 MHz, $(CD_3)_2CO$): δ = 148.1 (s, 2C, C3/5), 145.6 (s, br, 2C, C2/6), 127.3 (q, 3C, $^1J(^{13}C,^{11}B)$ = 67 Hz, CN), 116.0 (q, 1C, $^2J(^{13}C,^1H)$ = 6.3 Hz, C1), 114.4 (q, 1C, $^1J(^{13}C,^{11}B)$ = 56 Hz, C4), 7.3 ppm (q, 1C, $^1J(^{13}C,^1H)$ = 131 Hz, CH_3). $^{13}C\{^{13}B,^1H\}$ NMR (75.5 Hz, $(CD_3)_2CO$): δ = 148.1 (dddd, 2C, $^1J(^{19}F,^{13}C)$ = 243 Hz, $^{2/3}J(^{19}F,^{13}C)$ = 13.6 Hz, 11.6 Hz, $^4J(^{19}F,^{13}C)$ = 3.8 Hz, C3/5), 145.6 (dddd, 2C, $^1J(^{19}F,^{13}C)$ = 243 Hz, $^{2/3/4}J(^{19}F,^{13}C)$ = 16.9 Hz, 6.5 Hz, 3.5 Hz, C2/6), 127.3 (t, 3C, $^4J(^{19}F,^{13}C)$ = 1.3 Hz, CN), 116.0 (tt, 1C, $^2J(^{19}F,^{13}C)$ = 19.4 Hz, $^3J(^{19}F,^{13}C)$ = 1.2 Hz, C1), 114.4 (tt, 1C, $^2J(^{19}F,^{13}C)$ = 23.9 Hz, $^3J(^{19}F,^{13}C)$ = 1.7 Hz, C4), 7.3 ppm (tt, 1C, $^3J(^{19}F,^{13}C)$ = 3.5 Hz, $^4J(^{19}F,^{13}C)$ = 2.2 Hz, CH_3). NMR spectroscopic data for the side product K**B8b**: ^{11}B NMR (160.5 MHz, $(CD_3)_2CO$): δ = −33.8 ppm (s, 1B). ^{19}F NMR (470.6 MHz, $(CD_3)_2CO$): δ = −109.2 (s, 1F, F2), −130.4 (m, 1F, F4), −140.5 (d, 1F, $^{2/3}J(^{19}F,^{19}F)$ = 20.6 Hz, F6), −169.6 ppm (ddd, 1F, $^{2/3}J(^{19}F,^{19}F)$ = 21.0 Hz (2×), 11.9 Hz, F5). ^1H NMR (500.1 MHz, $(CD_3)_2CO$): δ = 2.14 ppm (dd, 3H, $^4J(^{19}F,^1H)$ = 2.2 Hz, 1.3 Hz). $^{13}C\{^{19}F\}$ NMR (125.8 MHz, $(CD_3)_2CO$): δ = 158.0 (m, 1C, C2), 151.1 (s, 1C, C4), 149.6 (q, 1C, $^3J(^{13}C,^1H)$ = 4.3 Hz, C6), 137.5 (s, 1C, C5), 127.5 (q, 3C, $^1J(^{13}C,^{11}B)$ = 67 Hz, CN), 110.7 (m, 1C, C1), 7.0 ppm (q, 1C, $^1J(^{13}C,^1H)$ = 131 Hz, CH_3) [the signal of C3 was not observed]. [In the $^{13}C\{^{13}B,^1H\}$ and $^{13}C\{^1H\}$ NMR spectra the signals of anion **B8b** were not observed.] Elemental analysis: calculated (%) for $C_{10}H_3BF_4KN_3$, C 41.27, H 1.04, N 14.44; found, C 40.88, H 1.11, N 14.10. (−)-ESI-MS, m/z, $[(CH_3C_6F_4)\{B(CN)_3\}]^-$: calculated 252.04 (100.0%), 251.04 (24.8%), 253.04 (10.8%); found 252.04 (100.0%), 251.04 (23.8%), 253.04 (10.3%).

K[1-F$_3$C-4-{(NC)$_3$B}-2,3,5-F$_3$-C$_6$H] (K**B9**): Method A was employed for the preparation of K**B9** using 2,3,4,5-tetrafluorobenzotrifluoride (ca. 0.5 mL, 3.4 mmol) and K_2**1** (300 mg, 1.80 mmol). The reaction mixture was stirred at room temperature for 4 days. Yield: 460 mg (1.41 mmol, 78%). The product contained ca. 7% of other isomers. ^{11}B NMR (160.5 MHz, $(CD_3)_2CO$): δ = −33.8 ppm (s, 1B). ^1H NMR (500.1 MHz, $(CD_3)_2CO$): δ = 7.24 ppm (m, 1H).

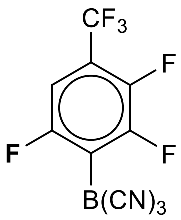

B9

$^1H\{^{11}B\}$ NMR (500.1 MHz, $(CD_3)_2CO$): δ = 7.24 ppm (dddq, 1H, $J(^{19}F,^1H)$ = 8.7 Hz, 4.9 Hz, 2.3 Hz, 0.6 Hz). ^{19}F NMR (470.6 MHz, $(CD_3)_2CO$): δ = −61.9 (dm, 3F, $^4J(^{19}F,^1H)$ = 12.8 Hz, CF_3), −103.7 (s, 1F, F5), −124.1 (s, 1F, F3), −147.5 ppm (m, 1F, F2). $^{13}C\{^1H\}$ NMR (125.8 MHz, $(CD_3)_2CO$): δ = 160.1 (ddd, 1C, $^1J(^{19}F,^{13}C)$ = 246 Hz, $^{3/4}J(^{19}F,^{13}C)$ ≈ 11.5 Hz, 3.5 Hz, C5), 153.3 (ddd, 1C, $^1J(^{19}F,^{13}C)$ = 249 Hz, $^2J(^{19}F,^{13}C)$ ≈ $^2J(^{19}F,^{13}C)$ ≈ 12 Hz, C3), 145.4 (dm,

br, 1C, $^{1}J(^{19}F,^{13}C)$ = 253 Hz, C2), 126.8 (q, 3C, $^{1}J(^{13}C,^{11}B)$ = 68 Hz, CN), 122.7 (qddd, 1C, $^{1}J(^{19}F,^{13}C)$ = 272 Hz, $^{3/4}J(^{19}F,^{13}C)$ = 4.3 Hz, 2.7 Hz, 0.9 Hz, CF_3), 122.5 (m, 1C, C4), 119.3 (qddd, 1C, $^{2}J(^{19}F,^{13}C)$ = 34.5 Hz, $^{2/3}J(^{19}F,^{13}C)$ = 11.1 Hz, 10.5 Hz, 1.9 Hz, C1), 109.3 ppm (dm, 1C, $^{2}J(^{19}F,^{13}C)$ = 31.7 Hz, C6). $^{13}C\{^{13}B,^{1}H\}$ NMR (75.5 Hz, $(CD_3)_2CO$): δ = 160.2 (ddd, 1C, $^{1}J(^{19}F,^{13}C)$ = 246 Hz, $^{3/4}J(^{19}F,^{13}C)$ ≈ 11.3 Hz, 3.3 Hz, C5), 153.3 (ddd, 1C, $^{1}J(^{19}F,^{13}C)$ = 249 Hz, $^{2}J(^{19}F,^{13}C)$ ≈ 13.3 Hz, 12.2 Hz, C3), 145.4 (ddm, 1C, $^{1}J(^{19}F,^{13}C)$ = 253 Hz, $^{2}J(^{19}F,^{13}C)$ ≈ 18 Hz, C2), 126.8 (s, 3C, CN), 122.7 (qm, 1C, $^{1}J(^{19}F,^{13}C)$ = 272 Hz, CF_3), 122.4 (ddm, 1C, $^{2}J(^{19}F,^{13}C)$ = 27.7 Hz, 22.0 Hz, C4), 119.3 (qddd, 1C, $^{2}J(^{19}F,^{13}C)$ = 34.5 Hz, $^{2/3}J(^{19}F,^{13}C)$ ≈ 10.8 Hz (2×), 1.9 Hz, C1), 109.3 ppm (ddq, 1C, $^{2}J(^{19}F,^{13}C)$ = 31.7 Hz, $^{3}J(^{19}F,^{13}C)$ ≈ 4.5 Hz (2×), C6). Elemental analysis: calculated (%) for $C_{10}HBF_6KN_3$, C 36.73, H 0.31, N 12.85; found, C 36.71, H 0.36, N 13.06. (−)-ESI-MS, m/z, $[(CF_3C_6F_3H)B(CN)_3]^-$: calculated 288.02 (100.0%), 287.02 (24.8%), 289.02 (10.8%); found 288.02 (100.0%), 287.02 (24.7%), 289.02 (10.4%).

K[1-CH$_3$O-4-{(NC)$_3$B}-C$_6$F$_4$] (K**B10a**) and **K[1-CH$_3$O-3-{(NC)$_3$B}-C$_6$F$_4$]** (K**B10b**):

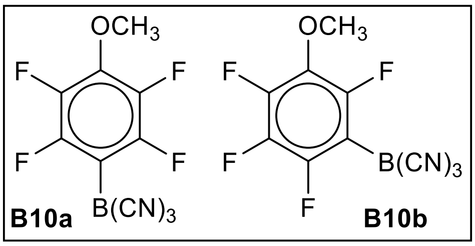

Method A was employed for the preparation of K**B10a** and K**B10b** using 2,3,4,5,6-pentafluoroaniline (ca. 0.6 mL, 4.2 mmol) and K$_2$**1** (300 mg, 1.80 mmol). The reaction mixture was stirred at 60 °C overnight. Yield: 450 mg (1.47 mmol, 82%). The product was a mixture of K**B10a** and K**B10b** (ratio 1:1). NMR spectroscopic data for K**B10a**: ^{11}B NMR (160.5 MHz, $(CD_3)_2CO$): δ = −33.8 ppm (s, 1B). ^{19}F NMR (470.6 MHz, $(CD_3)_2CO$): δ = −133.8 (m, 2F, F3/5), −160.2 ppm (dd, 2F, $^{2/3}J(^{19}F,^{19}F)$ = 21.0 Hz, 9.9 Hz, F2/6). ^{1}H NMR (500.1 MHz, $(CD_3)_2CO$): δ = 4.05 ppm (m, 3H). $^{13}C\{^{1}H\}$ NMR (125.8 MHz, $(CD_3)_2CO$): δ = 148.7 (dddd, 2C, $^{1}J(^{19}F,^{13}C)$ = 242 Hz, $^{3/4}J(^{19}F,^{13}C)$ = 14 Hz, 11 Hz, 4 Hz, C3/5), 141.4 (dm, br, 2C, $^{1}J(^{19}F,^{13}C)$ = 246 Hz, C2/6), 138.3 (tt, 1C, $^{2}J(^{19}F,^{13}C)$ = 12.2 Hz, $^{3}J(^{19}F,^{13}C)$ = 8.1 Hz, C1), 127.1 (q, 3C, $^{1}J(^{13}C,^{11}B)$ = 67 Hz, CN), 111.1 (m, 1C, C4), 62.7 ppm (t, 1C, $^{4}J(^{19}F,^{13}C)$ = 3 Hz, OCH_3). $^{13}C\{^{19}F\}$ NMR (125.8 MHz, $(CD_3)_2CO$): δ = 148.7 (s, 2C, C3/5), 141.4 (s, br, 2C, C2/6), 138.3 (s, 1C, C1), 127.1 (q, 3C, $^{1}J(^{13}C,^{11}B)$ = 67 Hz, CN), 111.1 (q, 1C, $^{1}J(^{13}C,^{11}B)$ = 57 Hz, C4), 62.6 ppm (q, 1C, $^{1}J(^{13}C,^{1}H)$ = 146 Hz, OCH_3). NMR spectroscopic data for K**B10b**: ^{11}B NMR (160.5 MHz, $(CD_3)_2CO$): δ = −33.8 ppm (s, 1B). ^{19}F NMR (470.6 MHz, $(CD_3)_2CO$): δ = −124.6 (s, 1F, F2), −133.6 (m, 1F, F4), −153.1 (dm, 1F, $^{3}J(^{19}F, ^{19}F)$ ≈ 20 Hz, F6), −167.0 ppm (ddd, 1F, $^{3/5}J(^{19}F, ^{19}F)$ ≈ 22 Hz, 20 Hz, 10 Hz, F5). ^{1}H NMR (500.1 MHz, $(CD_3)_2CO$): δ = 3.93 ppm (m, 3H). $^{13}C\{^{1}H\}$ NMR (125.8 MHz, $(CD_3)_2CO$): δ = 153.3 (ddm,

1C, $^{1}J(^{19}F,^{13}C)$ = 244 Hz, $^{3/4}J(^{19}F,^{13}C)$ = 13.5 Hz, C2), 148.2 (dm, 1C, $^{1}J(^{19}F,^{13}C)$ = 242 Hz, C4), 145.3 (dddd, 1C, $^{1}J(^{19}F,^{13}C)$ = 247 Hz, $^{2/3}J(^{19}F,^{13}C)$ = 11.7 Hz, 7.9 Hz, 5.9 Hz, C6), 137.8 (dm, br, $^{1}J(^{19}F,^{13}C)$ ≈ 247 Hz, 1C, C5), 134.5 (m, 1C, C1), 127.2 (q, 3C, $^{1}J(^{13}C,^{11}B)$ = 67 Hz, CN), 109.9 (m, 1C, C3), 62.3 ppm (q, 1C, $^{4}J(^{19}F,^{13}C)$ = 3.6 Hz, OCH_3). $^{13}C\{^{19}F\}$ NMR (125.8 MHz, $(CD_3)_2CO$): δ = 153.3 (s, 1C, C2), 148.2 (s, br, 1C, C4), 145.3 (s, 1C, C6), 137.8 (s, 1C, C5), 134.5 (s, 1C, C1), 127.2 (q, 3C, $^{1}J(^{13}C,^{11}B)$ = 67 Hz, CN), 109.9 (q, 1C, $^{1}J(^{13}C,^{11}B)$ = 56 Hz, C3), 62.3 ppm (q, 1C, $^{1}J(^{13}C,^{1}H)$ = 147 Hz, OCH_3). Elemental analysis: calculated (%) for $C_{10}H_3BF_4KN_3O$, C 39.12, H 0.98, N 13.69; found, C 38.59, H 0.89, N 15.15. (−)-ESI-MS, m/z, $[(CH_3OC_6F_4)B(CN)_3]^-$: calculated 268.03 (100.0%), 267.03 (24.8%), 269.03 (10.8%); found 268.03 (100.0%), 267.03 (25.5%), 269.03 (10.9%). Crystals of K**B10a** suitable for an X-ray diffraction study were obtained from an *iso*propanol solution by slow evaporation of the solvent.

K[1-NC-4-{(NC)$_3$B}-C$_6$F$_4$] (K**B11a**): <u>Method A</u> was employed for the preparation of K**B11a** using 2,3,4,5,6-pentafluorobenzonitrile (ca. 0.45 mL, 3.6 mmol) and K_2**1** (300 mg, 1.80 mmol). The arene was added at −100 °C and slowly warmed up to room temperature. The dark raw product was a mixture of K**B11a**, K[1-NC-2-{(NC)$_3$B}-C$_6$F$_4$] (K**B11b)** and K[B(CN)$_4$] (ratio 2.5:1.5:1.0). Yield: ca. 350 mg. Upon slow addition of CH_2Cl_2 to the THF layers several fractions were obtained which resulted in an enrichment of the main and the side product, respectively. NMR spectroscopic data for K**B11a**: ^{11}B NMR (160.5 MHz, $(CD_3)_2CO$): δ = −33.8 ppm (tt, 1B, $^{3}J(^{19}F,^{11}B)$ = 7.4 Hz, $^{4}J(^{19}F,^{11}B)$ = 2.7 Hz). ^{19}F NMR (470.6 MHz, $(CD_3)_2CO$): δ = −129.8 (m, 2F, F3/5), −136.8 (m, 2F, F2/6). $^{13}C\{^{1}H\}$ NMR (125.8 MHz, $(CD_3)_2CO$): δ = 148.2 (dddd, 2C, $^{1}J(^{19}F,^{13}C)$ = 246 Hz, $^{2/3/4}J(^{19}F,^{13}C)$ ≈ 11 Hz (2×), 4 Hz, C3/5), 147.6 (dm, 2C, $^{1}J(^{19}F,^{13}C)$ = 259 Hz, 2/6), 126.0 (q, 3C, $^{1}J(^{13}C,^{11}B)$ = 68 Hz, B–CN), 108.6 (t, 1C, $^{3}J(^{19}F,^{13}C)$ = 3.9 Hz, Ph–CN), 93.8 ppm (tt, 1C, $^{2}J(^{19}F,^{13}C)$ = 17.4 Hz, $^{3}J(^{19}F,^{13}C)$ = 3.2 Hz, C1) [the signal of C4 is covered by the signal of the B(CN)$_3$ group]. $^{13}C\{^{19}F\}$ NMR (125.8 MHz, $(CD_3)_2CO$): δ = 148.2 (s, 2C, C3/5), 147.6 (m, 2C, C2/6), 126.0 (q, 3C, $^{1}J(^{13}C,^{11}B)$ = 68 Hz, B–CN), 108.7 (s, 1C, Ph–CN), 93.8 ppm (s, 1C, C1) [the signal of C4 is covered by the signal of the B(CN)$_3$ group]. NMR spectroscopic data for the side product K**B11b**: ^{11}B NMR (160.5 MHz, $(CD_3)_2CO$): δ = −32.4 ppm (dm, 1B, $^{3}J(^{19}F,^{11}B)$ ≈ 7.2 Hz). ^{19}F NMR (470.6 MHz, $(CD_3)_2CO$): δ = −127.9 (m, 1F, F3), −133.8 (m, 1F, F6), −149.0 (m, 1F, F4), −156.8 ppm (ddd, 1F, $^{3}J(^{19}F,^{19}F)$ = 19.8 Hz (2×), $^{4}J(^{19}F,^{19}F)$ = 5.6 Hz, F5).

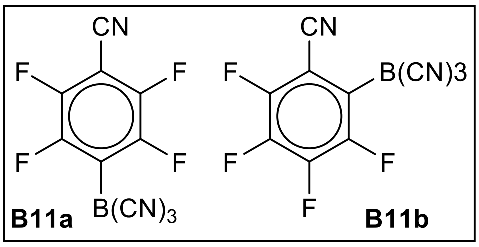

$^{13}C\{^1H\}$ NMR (125.8 MHz, $(CD_3)_2CO$): δ = 151.2 (dm, 1C, $^1J(^{19}F,^{13}C)$ = 258 Hz, C6), 149.9 (ddm, 1C, $^1J(^{19}F,^{13}C)$ = 246 Hz, $^2J(^{19}F,^{13}C)$ = 10.0 Hz, C3), 144.4 (dm, 1C, $^1J(^{19}F,^{13}C)$ ≈ 262 Hz, C4), 140.5 (ddd, 1C, $^1J(^{19}F,^{13}C)$ = 253 Hz, $^2J(^{19}F,^{13}C)$ = 15.9 Hz, 13.1 Hz, $^3J(^{19}F,^{13}C)$ = 4.4 Hz, C5), 126.4 (q, 3C, $^1J(^{13}C,^{11}B)$ = 68 Hz, B–CN),111.6 (s, br, 1C, Ph–CN), 101.9 ppm (dd, 1C, $^2J(^{19}F,^{13}C)$ ≈ 11 Hz (2×), C1) [the signal of C2 is covered by the signal of the $B(CN)_3$ group]. $^{13}C\{^{19}F\}$ NMR (125.8 MHz, $(CD_3)_2CO$): δ = 151.2 (s, br, 1C, C6), 149.9 (s, 1C, C3), 144.4 (s, br, 1C, C4), 140.5 (s, 1C, C5), 126.4 (q, 3C, $^1J(^{13}C,^{11}B)$ = 68 Hz, B–CN), 111.6 (s, br, 1C, Ph-CN), 101.9 ppm (s, 1C, C1) [the signal of C2 is covered by the signal of the $B(CN)_3$ group]. (−)-ESI-MS, m/z, $[(NCC_6F_4)B(CN)_3]^-$: calculated 263.02 (100.0%), 262.02 (24.8%), 264.02 (10.8%); found 263.02 (100.0%), 262.02 (24.7%), 264.02 (10.6%). Crystals of K**B11a** suitable for an X-ray diffraction study were obtained by slow diffusion of hexane in an ethyl acetate solution.

5. Table of Experimental and Calculated Chemical Shifts

Table S1. Selected experimental and calculated[a] chemical shifts[b] of the tricyanoborates.

anion		**B1**	**B2a**	**B2b**	**B2c**	**B3a**	**B3b**	**B3c**	**B3d**	**B4a**	**B4b**	**B4c**	**B5**	**B4d**	**B6a**[c]	**B6b**
$\delta(^{11}B)$	B	−28.9	−30.8	−29.1	−29.1	−31.0	−33.6	−31.0	−29.3	−33.7	−31.2	−31.2	−33.7	−31.1	−33.9	−34.7
		−33.1	*−34.9*	*−33.6*	*−33.4*	*−35.0*	*−37.6*	*−35.1*	*−33.5*	*−37.6*	*−35.1*	*−35.3*	*−37.6*	*−34.7*	*−37.8*	*−37.3*
$\delta(^{19}F/^{1}H)$	X2	7.5	−105.1	[d]	7.5	−132.5	−101.4	−111.7	7.0	−127.1	−137.2	−106.7	−132.7	−113.4	−132.1	−133.8
		8.1	*−119.7*	*7.8*	*8.0*	*−146.8*	*−113.7*	*−127.9*	*7.5*	*−141.5*	*−153.2*	*−122.5*	*−146.6*	*−136.3*	*−146.0*	*−157.7*
	X3	7.3	7.0	−116.1	7.0	−142.3	6.8	7.0	−112.7	−145.6	−136.7	7.1	−141.8	7.1	−165.4	−133.8
		7.2	*6.9*	*−141.9*	*6.9*	*−167.5*	*6.6*	*6.7*	*−139.4*	*−171.3*	*−162.8*	*6.6*	*−168.5*	*7.3*	*−192.9*	*−152.7*
	X4	7.2	7.3	6.9	−118.6	7.1	7.3	7.0	6.8	7.2	7.1	−137.8	7.3	-	−158.5	-
		7.1	*7.1*	*6.7*	*−147.6*	*6.8*	*7.0*	*6.7*	*6.4*	*6.7*	*6.5*	*−167.5*	*6.6*	-	*−188.7*	-
	X5	7.3	7.1	[d]	7.0	7.1	6.8	−122.1	−112.7	6.9	−117.8	−146.5	−141.8	−113.4	−165.4	−133.8
		7.2	*7.0*	*7.2*	*6.9*	*6.9*	*6.6*	*−148.9*	*139.4*	*6.5*	*−145.1*	*−173.7*	*−168.5*	*−136.3*	*−192.9*	*−157.7*
	X6	7.5	7.5	[d]	7.5	7.3	−101.4	7.2	7.0	−106.8	7.0	7.3	−132.7	7.1	−132.1	−133.8
		8.1	*8.1*	*7.7*	*8.0*	*7.8*	*−113.7*	*7.8*	*7.5*	*−118.2*	*7.6*	*7.8*	*−146.6*	*7.3*	*−146.0*	*−152.7*
$\delta(^{13}C)$	CN	129.6	128.5	129.1	129.5	128.1	128.2	128.0	128.6	127.5	127.5	127.8	127.0	128.3	126.7	127.4
		134.0	*132.4*	*133.4*	*133.8*	*130.4*	*131.9*	*131.9*	*132.9*	*131.3*	*131.4*	*131.7*	*130.8*	*134.6*	*130.7*	*133.2*
	C1	140.6	126.2	144.1	136.5	n.d.	n.d.	128.8	146.4	116.4	131.0	123.4	118.3	127.5	111.6	116.7
		155.2	*140.4*	*158.9*	*150.1*	*144*	*128.3*	*143.3*	*161.1*	*131.0*	*145.8*	*137.0*	*133.2*	*134.1*	*125.5*	*123.6*
	C2	133.3	166.3	119.3	135.0	153.5	166.1	162.2	115.4	152.6	149.7	160.9	148.3	162.2	148.4	148.2
		139.6	*176.6*	*124.5*	*140.7*	*163.2*	*176.2*	*171.9*	*120.1*	*161.4*	*158.8*	*170.3*	*157.2*	*171.5*	*157.2*	*156.9*
	C3	128.3	115.1	163.4	115.0	151.1	111.6	116.6	163.6	147.9	150.6	105.6	146.5	120.6	137.9	148.2
		130.0	*117.1*	*172.2*	*115.9*	*159.8*	*112.9*	*117.2*	*171.7*	*156.3*	*158.6*	*106.0*	*154.1*	*124.5*	*145.1*	*156.9*
	C4	127.2	130.0	113.9	162.9	116.9	130.5	115.9	102.4	117.2	105.0	150.1	106.1	127.5	141.1	116.7
		127.0	*129.0*	*112.9*	*170.9*	*116.1*	*128.8*	*114.6*	*101.3*	*115.3*	*103.5*	*157.0*	*104.2*	*134.1*	*147.5*	*123.6*
	C5	128.3	124.4	130.2	115.0	125.0	111.6	159.4	163.6	111.5	158.3	147.2	146.5	162.2	137.9	148.2
		130.0	*125.2*	*130.4*	*115.9*	*124.6*	*112.9*	*167.8*	*171.7*	*111.2*	*166.0*	*154.6*	*154.1*	*171.5*	*145.1*	*156.9*
	C6	133.3	135.4	129.1	135.0	130.2	166.1	120.9	115.4	160.8	115.6	122.1	148.3	120.6	148.4	148.2
		139.6	*141.9*	*135.8*	*140.7*	*135.8*	*176.2*	*126.9*	*120.1*	*171.5*	*120.7*	*128.3*	*157.2*	*124.5*	*157.2*	*156.9*

[a] B3LYP/6-311++G(2d,p) using geometries calculated at the B3LYP/6-311++G(2,p) level of theory; calculated values in italics; mean values where applicable. [b] Chemical shifts in ppm. [c] Literature values.[10] [d] A definite assignment even with the aid of calculated chemical shifts was not possible.

Table S1 continued.

anion		B11a	B7	B8a	B10a	B11b	B11c [e]	B8b	B10b	B9	BP2	BP1		Py1	Py2	Py3
$\delta(^{11}B)$	B	−33.8	−33.8	−33.8	−33.8	−32.4		−33.8	−33.8	−33.8	−33.7	−33.7	B4	−33.9	−33.9	−33.9
		−37.6	*−37.6*	*−37.7*	*−37.7*	*−36.5*	*−37.9*	*−37.8*	*−37.8*	*−37.7*	*−37.4*	*−37.6*		*−37.7*	*−37.4*	*−37.1*
													B2/6	-	−30.9	−30.0
														-	*−34.2*	*−33.3*
$\delta(^{19}F)^c$	X2	−136.8	−144.3	−146.7	−160.2	-		−109.2	−124.6	−147.5	−141.7	−141.6		−96.0	-	-
		−158.6	*−166.7*	*−173.7*	*−186.4*	-	*−109.4*	*−118.3*	*−134.7*	*−169.1*	*−165.4*	*−167.1*		*−118.0*	-	-
	X3	−129.8	−130.4	−134.1	−133.8	−127.9	-	-	-	−124.1	−131.7	−131.4		−134.7	−108.3	−107.5
		−143.2	*−144.5*	*−148.2*	*−149.5*	*−143.6*	-	-	-	*−133.5*	*−150.8*	*−145.3*		*−149.3*	*−123.6*	*−125.2*
	X4	-	-	-	-	−149.0		−130.4	−133.6	-	-	-		-	-	-
		-	-	-	-	*−174.9*	*−119.4*	*−144.8*	*−148.5*	-	-	-		-	-	-
	X5	−129.8	−130.4	−134.1	−133.8	−156.8		−169.6	−167.0	−103.7	−131.7	−131.4		−134.7	−132.4	−107.5
		−143.2	*−144.5*	*−148.2*	*−146.0*	*−187.7*	*−192.6*	*−195.9*	*−193.3*	*−121.0*	*−150.8*	*−145.3*		*−149.3*	*−152.6*	*−125.2*
	X6	−136.8	−144.3	−146.7	−160.2	−133.8		−140.5	−153.1	7.24	−141.7	−141.6		−96.0	−94.7	-
		−158.6	*−166.7*	*−173.7*	*−186.4*	*−156.4*	*−155.7*	*−172.1*	*−182.6*	*6.9*	*−165.4*	*−167.1*		*−118.0*	*−121.2*	-
$\delta(^{13}C)$	CN	126.0	126.4	127.3	127.1	126.4		127.5	127.2	126.8	126.7	126.7	B4-CN	125.8	[d]	128.3
		129.6	*130.0*	*131.1*	*131.0*	*130.0*	*130.0*	*131.4*	*131.2*	*130.5*	*131.7*	*130.3*		*129.8*	*132.6*	*135.2*
													B2/6-CN	-	[d]	128.3
														-	*133.1*	*135.7*
	C1	93.8	108.8	116.0	138.3	101.9		110.7	134.5	119.3	107.1	103.6		-	-	-
		93.4	*110.7*	*115.2*	*141.3*	*108.8*	*91.1*	*111.9*	*138.3*	*121.4*	*112.5*	*106.0*		-	-	-
	C2	147.6	144.5	145.6	141.4	n.d.		158.0	153.3	145.4	144.6	144.6		144.0	142.8	146.4
		156.2	*152.0*	*153.0*	*150.1*	*144.1*	*170.1*	*168.5*	*164.5*	*153.5*	*152.7*	*151.8*		*150.9*	*153.6*	*154.2*
	C3	148.2	148.8	148.1	148.7	149.9		n.d.	109.3	153.3	148.5	148.6		144.0	161.9	164.7
		157.0	*157.4*	*157.2*	*157.5*	*157.0*	*127.1*	*123.9*	*124.1*	*163.6*	*157.2*	*157.5*		*152.6*	*170.7*	*173.6*
	C4	n.d.	123.6	114.4	111.1	144.4		151.1	148.2	122.5	120.3	121.1		133.5	[d]	118.4
		141.6	*138.3*	*129.3*	*126.2*	*151.3*	*167.3*	*159.9*	*157.0*	*137.1*	*129.9*	*136.7*		*147.3*	*134.8*	*123.2*
	C5	148.2	148.8	148.1	148.7	140.5		137.5	137.8	160.1	148.5	148.6		144.0	146.3	164.7
		157.0	*157.4*	*157.2*	*157.5*	*146.2*	*144.9*	*145.3*	*145.5*	*168.0*	*157.2*	*157.5*		*152.6*	*154.8*	*173.6*
	C6	147.6	144.5	145.6	141.4	151.2		149.6	145.3	109.3	144.6	144.6		144.0	147.9	146.4
		156.2	*152.0*	*153.0*	*150.1*	*159.3*	*159.9*	*155.7*	*151.8*	*110.5*	*152.7*	*151.8*		*150.9*	*153.9*	*154.2*
	C_{Gr}	108.6	122.1	7.3	62.6	111.6		7.0	62.3	122.7	-	[f]		-	-	-
		114.2	*135.2*	*7.8*	*62.1*	*115.4*	*114.2*	*7.4*	*61.7*	*135.8*	-	[f]		-	-	-

[e] **B11c** = $[1\text{-}H_3C\text{-}3\text{-}\{(NC)_3B\}\text{-}C_6F_4]^-$. [f] Data of the second Ph-ring: C1‘: 105.8 (*114.4*), C2‘/6‘: 145.4 (*154.1*), C3‘/C5‘: 138.9 (*146.0*), C4‘: 143.3 (*149.5*); F2‘/6‘: −139.8 (*−155.4*), F3‘/5‘: −163.3 (*−188.7*), F4‘: −153.2 (*−180.6*).

Table S1 continued.

anion		N1	N2	N3
δ(¹¹B)	B	−33.6	−33.6	−33.6
		−37.5	*−37.2*	*−37.2*
δ(¹⁹F)	F1	−107.3	−108.8	−104.6
		−120.9	*−127.6*	*−118.4*
	F3	−127.3	−130.1	−127.6
		−131.7	*−147.0*	*−143.1*
	F4	−153.3	−152.1	−154.5
		−177.3	*−175. 9*	*−182.8*
	F5	−149.4	−108.8	−154.5
		−171.5	*−127.6*	*−182.8*
	F6	−157.3	-	−127.6
		−186.5	-	*−143.1*
	F7	−159.6	−130.1	-
		−189.1	*−147.0*	-
	F8	−146.3	−152.1	−104.6
		−163.0	*−175.9*	*−118.4*
δ(¹³C)	CN	127.0	127.3	n.d.
		130.4	*132.6*	*132.6*
	C1	[d]	154.2	n.d.
		162.3	*161.9*	*163.6*
	C2	116.8	116.7	n.d.
		132.4	*125.1*	*122.9*
	C3	[d]	149.7	n.d.
		162.5	*159.4*	*160.6*
	C4	[d]	140.9	n.d.
		148.4	*149.5*	*148.4*
	C4a	112.2	112.9	n.d.
		115.4	*117.1*	*119.6*
	C5	[d]	154.2	n.d.
		149.4	*161.9*	*148.4*
	C6	[d]	116.7	n.d.
		146.5	*125.1*	*160.6*
	C7	[d]	149.7	n.d.
		145.1	*159.4*	*122.9*
	C8	[d]	140.9	n.d.
		151.3	*149.5*	*163.6*
	C8a	108.9	112.9	n.d.
		112.9	*117.1*	*115.0*

6. Crystal Structure Determination

All crystal data were collected on a Bruker X8-Apex II diffractometer with a CCD area detector and multi-layer mirror or graphite monochromated $Mo_{K\alpha}$ radiation. The structures were solved by intrinsic phasing methods (SHELXT).[11-12] Refinement is based on full-matrix least-squares calculations on F^2 (SHELXL).[12-13] All non-hydrogen atoms were refined anisotropically. Hydrogen atoms bonded to the phenyl rings of the anions were refined without any restraints. For all remaining CH moieties idealized bond lengths and angles were used and their isotropic displacement parameters were fixed to the equivalent isotropic displacement parameters of the respective parent carbon atom. The crystal of K_2**N2**·2EtOAc was a pseudo-merohedral twin with domains rotated by 179.9 ° around the real axis [−0.001 0.000 1.000]. The BASF parameter was refined to 50%. Calculations were carried out using the ShelXle graphical interface.[14] Molecular structure diagrams were drawn with the program Diamond 4.3.2.[15]

Crystallographic data have been deposited with the Cambridge Crystallographic Data Centre. Experimental details, crystal data, and CCDC numbers are collected in Table S2. These data can be obtained free of charge from The Cambridge Crystallographic Data Centre via www.ccdc.cam.ac.uk/data_request/cif.

Table S2. Crystal data and structure refinement details of the tricyanoborates.

compound	K**B2a**	K**B2b**	K**B3a**	K**B3c**	K**B3d**	$[Et_3NH]_2$**B4d**	K**B4b**	K**B4a**	$[Me_3NH]$**B4c**	$[Et_4N]_2$**B6b**
empirical formula	$C_{18}H_8B_2F_2K_2N_6$	$C_9H_4BFKN_3$	$C_9H_3BF_2KN_3$	$C_9H_3BF_2KN_3$	$C_9H_3BF_2KN_3$	$C_{24}H_{34}B_2F_2N_8$	$C_9H_2BF_3KN_3$	$C_9H_2BF_3KN_3$	$C_{12}H_{13}BF_3N_4$	$C_{28}H_{40}B_2F_4N_8$
M_W [g mol^{-1}]	446.12	223.06	241.05	241.05	241.05	494.21	259.05	259.05	281.07	586.30
colour	colourless	colourless	colourless	colourless	colourless	colourless	colourless	colourless	colourless	colourless
T [K]	100	100	100	100	100	100	100	100	100	100
crystal system	triclinic	monoclinic	triclinic	monoclinic	monoclinic	triclinic	monoclinic	monoclinic	orthorhombic	triclinic
space group	$P-1$	$P2_1/c$	$P-1$	$P2_1/n$	$P2_1/c$	$P-1$	$P2_1/c$	$P2_1/n$	$Ima2$	$P-1$
a [Å]	9.0844(6)	11.3175(10)	7.2218(4)	7.3664(11)	11.1415(11)	7.2310(18)	7.1368(4)	7.4212(4)	7.7462(5)	9.2925(5)
b [Å]	9.8593(6)	12.5441(11)	7.3375(4)	21.174(3)	12.3048(12)	10.113(3)	21.0081(11)	21.5366(12)	16.7938(10)	13.9870(7)
c [Å]	12.0751(8)	7.3312(6)	11.5902(7)	7.3900(11)	7.4382(7)	10.502(3	6.8083(3)	7.4276(4)	10.8073(7)	19.7322(10)
α [°]	91.429(2)		100.165(2)			98.148(9)				103.804(2)
β [°]	109.254(2)	100.954(4)	95.640(2)	117.188(4)	99.762(3)	105.678(9)	90.830(2)	118.496(2)		92.966(2)
γ [°]	90.945(2)		116.645(2)			106.755(8)				107.242(2)
V [Å^3]	1020.35(11)	1021.83(15)	529.22(5)	1025.3(3)	1004.97(17)	687.6(3)	1020.67(9)	1043.31(10)	1405.90(15)	2357.8(2)
Z	2	4	2	4	4	1	4	4	4	3
ρ_{calcd} [Mg m^{-3}]	1.452	1.450	1.513	1.562	1.593	1.194	1.686	1.649	1.328	1.239
μ [mm^{-1}]	0.499	0.498	0.500	0.516	0.527	0.083	0.539	0.527	0.110	0.092
F(000) [e]	448	448	240	480	480	262	512	512	580	930
θ range [°]	2.376–26.372	1.833–26.791	1.822–26.798	1.924–26.802	1.855–26.811	2.166–26.973	1.939–26.815	1.891–26.798	2.241–26.756	1.072–26.886
reflections collected	13952	13906	7270	8062	9084	9798	13961	14316	4838	31095
independent reflections [$I > 2\sigma(I)$]	3639	1792	2100	1765	1779	1980	1886	2035	1477	6261
R(int)	0.0311	0.0495	0.0195	0.0373	0.0481	0.0646	0.0367	0.0324	0.0250	0.0509
data / restraints / parameters	4180 / 0 / 303	2185 / 0 / 152	2258 / 0 / 145	2198 / 0 / 157	2139 / 0 / 157	2926 / 0 / 231	2183 / 0 / 162	2236 / 0 / 162	1597 / 1 / 140	10150 / 131 / 771
$R1$ [$I > 2\sigma(I)$]	0.0272	0.0369	0.0389	0.0336	0.0317	0.0458	0.0284	0.0272	0.0287	0.0624
$wR2$ (all data)	0.0677	0.0876	0.1059	0.0784	0.0835	0.1260	0.0647	0.0657	0.0686	0.1745
GOF on F^2	1.037	1.063	1.078	1.053	1.055	0.984	1.051	1.087	1.030	1.030
largest diff. peak / hole [e Å^{-3}]	0.327 / −0.252	0.329 / −0.324	1.575 / −0.429	0.315 / −0.238	0.374 / −0.355	0.325 / −0.328	0.399 / −0.202	0.382 / −0.219	0.179 / −0.136	0.746 / −0.312
CCDC number	1548900	1548901	1548902	1548903	1548904	1548893	1548906	1548905	1548895	1548894

Table S2 continued.

compound	K_2**BP2**·THF	KPy1	K_2**Py2**·$OC(CH_3)_2$	K_3**Py3**·3THF·1.04H_2O	K**B7**·THF	KB10a	KB11a	K_2**N2**·2EtOAc
empirical formula	$C_{26}H_{16}B_2F_8K_2N_6O_2$	$C_{16}B_2F_8K_2N_8$	$C_{14}H_6B_2F_3K_2N_7O$	$C_{52}H_{52.17}B_6F_4K_6N_{20}O_{8.01}$	$C_{56}H_{32}B_4F_{28}K_4N_{12}O_4$	$C_{10}H_3BF_4KN_3O$	$C_{20}B_2F_8K_2N_8$	$C_{12}H_8BF_3KN_3O_2$
M_W [g mol^{-1}]	696.27	556.06	445.08	1460.91	1668.57	307.06	604.10	333.12
colour	colourless	colourless	colourless	colourless	colourless	colourless	colourless	colourless
T [K]	100	100	100	100	100	100	100	100
crystal system	monoclinic	orthorhombic	tetragonal	monoclinic	monoclinic	orthorhombic	triclinic	monoclinic
space group	$C2$	$Pbca$	$I4_1/a$	$C2/c$	$P2_1/c$	$Pnma$	$P-1$	$P2_1/c$
a [Å]	28.487(4)	10.0487(6)	25.5118(15)	41.3012(11)	12.5972(7)	10.9629(9)	9.8453(7)	12.9371(9)
b [Å]	12.3676(16)	18.9832(12)	25.5118(15)	8.4531(2)	32.4417(17)	8.0873(7)	10.0627(7)	7.5514(5)
c [Å]	8.8225(11)	22.2949(13)	12.1914(8)	46.0735(11)	16.9137(9)	14.1771(10)	13.4136(10)	14.3323(10)
α [°]							87.223(3)	
β [°]	94.050(5)			113.7870(10)	92.820(3)		87.467(3)	93.205(3)
γ [°]							66.895(2)	
V [Å^3]	3100.6(7)	4252.9(4)	7934.8(11)	14718.9(6)	6903.8(6)	1256.95(17)	1220.40(15)	1397.98(17)
Z	4	8	16	8	4	4	2	4
ρ_{calcd} [Mg m^{-3}]	1.492	1.737	1.490	1.319	1.605	1.623	1.644	1.583
μ [mm^{-1}]	0.389	0.539	0.525	0.425	0.388	0.468	0.477	0.423
F(000) [e]	1400	2176	3552	5986	3328	608	592	672
θ range [°]	1.433–26.785	1.827–26.727	1.596–26.030	1.120–26.373	1.255– 26.372	2.348–26.791	2.202–26.372	2.847–26.372
reflections collected	21628	28164	31833	44601	89364	10502	16011	17851
independent reflections [$I > 2\sigma(I)$]	5996	3993	3088	11477	7469	1158	3659	2516
R(int)	0.0318	0.0258	0.0442	0.0285	0.1395	0.0522	0.0465	0.0751
data / restraints / parameters	6608 / 1 / 415	4508 / 0 / 325	3902 / 12 / 322	15069 / 33 / 944	14119 / 218 / 1047	1439 / 0 / 104	5004 / 0 / 361	2857 / 0 / 202
$R1$ [$I > 2\sigma(I)$]	0.0301	0.0575	0.0734	0.0457	0.0552	0.0362	0.0400	0.0500
$wR2$ (all data)	0.0611	0.1509	0.1519	0.1377	0.1311	0.0984	0.0802	0.1260
GOF on F^2	1.038	1.134	1.272	1.052	1.009	1.072	1.009	1.087
largest diff. peak / hole [e Å^{-3}]	0.272 / −0.172	1.293 / −0.546	1.210 / −0.857	1.075 / −0.505	0.544 / −0.435	0.495 / −0.270	0.355 / −0.278	0.920 / −0.478
CCDC number	1548896	1548910	1548898	1548899	1548907	1548908	1548909	1548897

7. Experimental and Calculated Bond Lengths

Table S3. Experimental and calculated[a] bond lengths[b] of the tricyanoborate anions.

anion	**B2a**	**B2b**	**B3a**	**B3c**	**B3d**	**B4a**	**B4b**	**B4c**	**B4d**	**B6a** [10]	**B6b** [c]
d(B–CN)	1.605(3)	1.601(3)	1.598(3)	1.598(3)	1.600(3)	1.600(2)	1.602(2)	1.601(3)	1.601(3)	1.607(2)	1.588(5)
	1.597(2)	1.604(3)	1.606(3)	1.615(3)	1.601(2)	1.604(2)	1.605(2)	1.601(3)	1.600(2)	1.605(2)	1.578(4)
	1.605(2)	1.607(3)	1.605(3)	1.600(3)	1.608(3)	1.606(2)	1.599(2)	1.598(4)	1.603(3)	1.599(2)	1.602(4)
	1.599	*1.599*	*1.598*	*1.598*	*1.598*	*1.599*	*1.597*	*1.598*	*1.604*	*1.597*	*1.603*
d(C–N)	1.145(2)	1.144(3)	1.142(3)	1.147(2)	1.145(2)	1.141(2)	1.143(2)	1.143(3)	1.145(2)	1.141(2)	1.128(4)
	1.146(2)	1.147(2)	1.145(3)	1.144(3)	1.144(2)	1.141(2)	1.142(2)	1.143(3)	1.146(2)	1.142(2)	1.143(4)
	1.147(2)	1.145(2)	1.145(3)	1.145(2)	1.148(2)	1.143(2)	1.145(2)	1.141(4)	1.144(2)	1.147(2)	1.145(4)
	1.157	*1.157*	*1.156*	*1.156*	*1.157*	*1.156*	*1.156*	*1.156*	*1.157*	*1.156*	*1.157*
d(B–C1)	1.612(2)	1.614(3)	1.606(3)	1.611(3)	1.616(2)	1.613(2)	1.618(2)	1.618(4)	1.612(3)	1.626(2)	1.623(3)
	1.638	*1.637*	*1.640*	*1.639*	*1.638*	*1.648*	*1.642*	*1.639*	*1.634*	*1.652*	*1.644*
d(C2–F2)	1.378(2)	-	1.352(3)	1.365(2)	-	1.346(2)	1.360(2)	1.364(3)	1.370(2)	1.345(2)	1.356(3)
	1.361	-	*1.351*	*1.360*	-	*1.346*	*1.350*	*1.356*	*1.370*	*1.344*	*1.354*
d(C3–F3)	-	1.373(2)	1.335(3)	-	1.361(2)	1.360(2)	1.352(2)	-	-	1.348(2)	1.350(3)
	-	*1.369*	*1.358*	-	*1.365*	*1.358*	*1.354*	-	-	*1.346*	*1.356*
d(C4–F4)	-	-	-	-	-	-	-	1.351(4)	-	1.343(2)	1.616(3) [d]
	-	-	-	-	-	-	-	*1.355*	-	*1.344*	*1.644*
d(C5–F5)	-	-	-	1.372(2)	1.372(2)	-	1.364(2)	1.353(4)	-	1.344(2)	1.352(3)
	-	-	-	*1.368*	*1.365*	-	*1.364*	*1.357*	-	*1.345*	*1.354*
d(C6–F6)	-	-	-	-	-	1.360(2)	-	-	-	1.351(2)	1.349(3)
	-	-	-	-	-	*1.356*	-	-	-	*1.344*	*1.356*
d(C1–C2)	1.381(2)	1.392(3)	1.383(3)	1.391(3)	1.399(2)	1.385(2)	1.385(2)	1.387(4)	1.374(3)	1.385(2)	1.373(4)
	1.394	*1.401*	*1.393*	*1.395*	*1.403*	*1.394*	*1.394*	*1.394*	*1.392*	*1.394*	*1.394*
d(C2–C3)	1.377(2)	1.385(3)	1.383(3)	1.378(3)	1.374(2)	1.381(2)	1.377(2)	1.379(5)	1.377(3)	1.383(2)	1.376(3)
	1.388	*1.386*	*1.390*	*1.387*	*1.384*	*1.394*	*1.389*	*1.389*	*1.387*	*1.393*	*1.391*
d(C3–C4)	1.389(3)	1.361(3)	1.371(4)	1.384(3)	1.377(3)	1.370(2)	1.376(2)	1.376(4)	1.396(2)	1.374(3)	1.380(4)
	1.394	*1.383*	*1.384*	*1.394*	*1.386*	*1.380*	*1.385*	*1.384*	*1.402*	*1.385*	*1.394*
d(C4–C5)	1.384(3)	1.375(3)	1.379(4)	1.374(4)	1.379(3)	1.388(2)	1.375(2)	1.380(5)	1.374(3)	1.370(3)	1.382(4)
	1.393	*1.397*	*1.394*	*1.383*	*1.386*	*1.393*	*1.385*	*1.386*	*1.392*	*1.387*	*1.394*
d(C5–C6)	1.388(2)	1.379(3)	1.387(3)	1.377(3)	1.380(3)	1.372(2)	1.377(2)	1.372(5)	1.377(3)	1..386(2)	1.374(3)
	1.396	*1.392*	*1.394*	*1.386*	*1.384*	*1.385*	*1.384*	*1.386*	*1.387*	*1.389*	*1.391*
d(C6–C1)	1.398(2)	1.397(3)	1.403(4)	1.397(3)	1.399(2)	1.393(2)	1.402(2)	1.396(4)	1.396(2)	1.387(3)	1.385(4)
	1.403	*1.405*	*1.403*	*1.402*	*1.403*	*1.399*	*1.403*	*1.402*	*1.402*	*1.397*	*1.394*

[a] B3LYP/6-311++G(d,p); calculated values in italics; mean values where applicable. [b] Bond lengths in Å. [c] Data of the second {$B(CN)_3$} moiety: *d*(B–CN) = 1.589(4), 1.603(4), 1.597(4) Å [*calc: 1.603 Å*]; *d*(C–N) = 1.134(4), 1.136(4), 1.145(3) Å [*calc: 1.157 Å*]; [d] *d*(C4–B).

Table S3 continued.

anion	B11a	B7	B10a	BP2 [f]
d(B–CN)	1.597(4)	1.602(5)	1.604(3)	1.595(4)
	1.609(4)	1.590(5)	1.589(3)	1.604(5)
	1.601(4)	1.586(5)	1.604(3)	1.599(5)
	1.600	*1.596*	*1.598*	*1.600*
d(C–N)	1.140(4)	1.137(5)	1.139(3)	1.138(4)
	1.142(3)	1.141(5)	1.139(3)	1.138(4)
	1.146(3)	1.138(5)	1.139(3)	1.142(4)
	1.155	*1.156*	*1.156*	*1.156*
d(C2–F2)	1.343(3)	1.342(5)	1.349(3)	1.343(3)
	1.342	*1.343*	*1.349*	*1.349*
d(C3–F3)	1.353(3)	1.344(4)	1.346(3)	1.350(3)
	1.342	*1.344*	*1.346*	*1.348*
d(C4–B)	1.626(3)	1.618(6)	1.621(4)	1.627(4)
	1.654	*1.653*	*1.650*	*1.649*
d(C5–F5)	1.349(3)	1.348(4)	1.346(3)	1.351(3)
	1.341	*1.345*	*1.347*	*1.349*
d(C6–F6)	1.341(3)	1.344(4)	1.349(3)	1.346(3)
	1.342	*1.343*	*1.352*	*1.348*
d(C1–C2)	1.386(4)	1.374(6)	1.368(3)	1.389(4)
	1.395	*1.391*	*1.392*	*1.392*
d(C2–C3)	1.374(3)	1.368(6)	1.391(3)	1.378(4)
	1.389	*1.395*	*1.393*	*1.392*
d(C3–C4)	1.387(4)	1.370(5)	1.386(3)	1.384
	1.397	*1.394*	*1.393*	*1.393*
d(C4–C5)	1.391(4)	1.382(5)	1.386(3)	1.391(4)
	1.399	*1.398*	*1.397*	*1.396*
d(C5–C6)	1.371(3)	1.372(6)	1.391(3)	1.380(4)
	1.385	*1.385*	*1.387*	*1.388*
d(C6–C1)	1.384(4)	1.377(6)	1.368(3)	1.386(4)
	1.398	*1.397*	*1.395*	*1.394*
d(C1-Gr.)	1.425(3)	1.504(6)	1.373(4)	1.481(4)
	1.423	*1.506*	*1.367*	*1.485*

		Py1	Py2	Py3
d(B–CN)	at C2	-	1.597(7)	1.590(3)
		-	1.590(7)	1.596(4)
		-	1.601(6)	1.600(4)
		-	*1.601*	*1.607*
	at C4	1.591(4)	1.580(37)	1.591(4)
		1.597(4)	1.594(31)	1.604(3)
		1.594(4)	1.593(32)	1.596(3)
		1.596	*1.602*	*1.606*
	at C6	-	-	1.589(3)
		-	-	1.597(3)
		-	-	1.597(3)
		-	-	*1.606*
d(C–N)	at C2	-	1.144(7)	1.148(2)
		-	1.135(7)	1.144(4)
		-	1.139(6)	1.137(4)
		-	*1.157*	*1.158*
	at C4	1.140(4)	1.139(31)	1.142(3)
		1.144(4)	1.137(27)	1.137(3)
		1.144(4)	1.133(24)	1.142(3)
		1.156	*1.157*	*1.157*
	at C6	-	-	1.140(3)
		-	-	1.146(3)
		-	-	1.144(2)
		-	-	*1.158*
d(C2–X2) [e]		1.340(4)	1.627(6)	1.620(4)
		1.345	*1.639*	*1.639*
d(C3–F3)		1.335(4)	1.366(5)	1.356(3)
		1.343	*1.353*	*1.363*
d(C4–B)		1.631(4)	1.728(26)	1.620(3)
		1.651	*1.646*	*1.642*
d(C5–F5)		1.345(4)	1.328(5)	1.374(3)
		1.343	*1.353*	*1.362*
d(C6–X6) [e]		1.337(4)	1.352(5)	1.617(4)
		1.346	*1.361*	*1.640*
d(N-C2)		1.310(4)	1.352(5)	1.3445(3)
		1.312	*1.349*	*1.347*
d(C2–C3)		1.377(5)	1.374(5)	1.390(2)
		1.387	*1.400*	*1.397*
d(C3–C4)		1.385(4)	1.385(6)	1.382(3)
		1.397	*1.401*	*1.396*
d(C4–C5)		1.387(4)	1.368(6)	1.386(4)
		1.394	*1.391*	*1.393*
d(C5–C6)		1.376(4)	1.378(5)	1.380(2)
		1.392	*1.387*	*1.400*
d(C6–N)		1.314(4)	1.296(5)	1.347(3)
		1.309	*1.300*	*1.345*

[e] X = F or B; [f] data for the second Ph ring: *d*(B–CN) = 1.601(4), 1.592(4), 1.612(4) Å [*calc: 1.600 Å*]; *d*(C–N) = 1.143(3), 1.142(4), 1.136(4) Å [*calc: 1.156 Å*]; *d*(C2'–F2') = 1.343(3) Å [*calc: 1.348 Å*]; *d*(C3'–F3') = 1.350(3) Å [*calc: 1.349 Å*]; *d*(C4'–B) = 1.625(4) Å [*calc: 1.649 Å*]; *d*(C5'–F5') = 1.349(3) Å [*calc: 1.348 Å*]; *d*(C6'–F6') = 1.341(3) Å [*calc: 1.349 Å*]; *d*(C1'–C2') = 1.389(4) Å [*calc: 1.394 Å*]; *d*(C2'–C3') = 1.383(4) Å [*calc: 1.388 Å*]; *d*(C3'–C4') = 1.394(4) Å [*calc. 1.396 Å*]; *d*(C4'–C5') = 1.380(4) Å [*calc: 1.393 Å*]; *d*(C5'–C6') = 1.385(4) Å [*calc: 1.392 Å*]; *d*(C6'–C1') = 1.386(4) Å [*calc: 1.392 Å*].

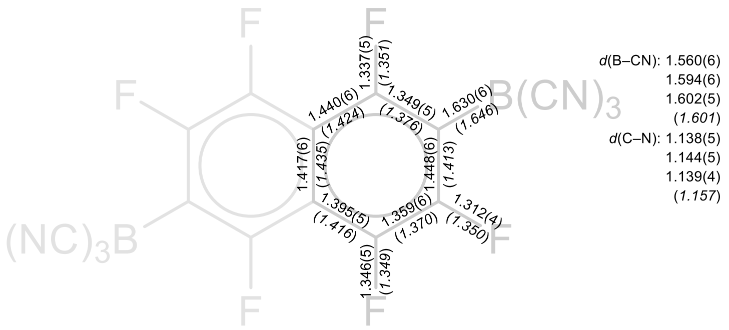

Figure S2: Experimental and calculated bond lengths [Å] of anion **N2**.

8. References

1 R. K. Harris, E. D. Becker, S. M. Cabral de Menezes, R. Goodfellow, P. Granger, *Pure Appl. Chem.*, 2001, **73**, 1795–1818.

2 G. R. Fulmer, A. J. M. Miller, N. H. Sherden, H. E. Gottlieb, A. Nudelman, B. M. Stoltz, J. E. Bercaw, K. I. Goldberg, *Organometallics*, 2010, **29**, 2176–2179.

3 W. Kohn, L. J. Sham, *Phys. Rev. A*, 1965, **140**, 1133–1138.

4 K. Wolinski, J. F. Hinton, P. Pulay, *J. Am. Chem. Soc.*, 1990, **112**, 8251–8260.

5 J. D. Kennedy, in *Multinuclear NMR* (Ed.: J. Mason), Plenum Press, New York, 1987, p. 221.

6 S. Berger, S. Braun, H.-O. Kalinowski, *NMR-Spektroskopie von Nichtmetallen - ^{19}F-NMR-Spektroskopie, Vol. 4*, Georg Thieme Verlag, Stuttgart, 1994.

7 M. J. Frisch, G. W. Trucks, H. B. Schlegel, G. E. Scuseria, M. A. Robb, J. R. Cheeseman, V. Barone, B. Mennucci, G. A. Petersson, H. Nakatsuji, M. Caricato, X. Xi, H. P. Hratchian, A. F. Izmaylov, J. Bloino, G. Zheng, J. L. Sonnenberg, M. Hada, M. Ehara, K. Toyota, R. Fukuda, J. Hasegawa, M. Ishida, T. Nakajima, Y. Honda, O. Kitao, H. Nakai, T. Vreven, J. J. A. Montgomery, J. E. Peralta, M. B. F. Ogliaro, J. J. Heyd, E. Brothers, K. N. Kudin, V. N. Staroverov, R. Kobayashi, J. Normand, K. Raghavachari, A. Rendell, J. C. Burant, S. S. Iyengar, J. Tomasi, M. Cossi, N. Rega, J. M. Millam, M. Klene, J. E. Knox, J. B. Cross, V. Bakken, C. Adamo, J. Jaramillo, R. Gomperts, R. E. Stratmann, O. Yazyev, A. J. Austin, R. Cammi, C. Pomelli, J. W. Ochterski, R. L. Martin, K. Morokuma, V. G. Zakrzewski, G. A. Voth, P. Salvador, J. J. Dannenberg, S. Dapprich, A. D. Daniels, Ö. Farkas, J. B. Foresman, J. V. Ortiz, J. Cioslowski, D. J. Fox, *Gaussian 09*, *Revision D.01*, Gaussian, Inc., Wallingford CT, USA, 2009.

8 W. L. F. Armarego, C. L. L. Chai, *Purification of Laboratory Chemicals*, 5 ed., Butterworth-Heinemann (Elsevier), 2003.

9 J. Landmann, F. Keppner, D. B. Hofmann, J. A. P. Sprenger, M. Häring, S. H. Zottnick, K. Müller-Buschbaum, N. V. Ignat'ev, M. Finze, *Angew. Chem.*, 2017, **129**, 2839–2843; *Angew. Chem. Int. Ed.* **2017**, *56*, 2795–2799.

10 J. Landmann, J. A. P. Sprenger, R. Bertermann, N. Ignat’ev, V. Bernhardt-Pitchougina, E. Bernhardt, H. Willner, M. Finze, *Chem. Commun.*, 2015, **51**, 4989–4992.

11 G. M. Sheldrick, *SHELXT, Program for Crystal Structure Solution*, Universität Göttingen, 2014.

12 G. M. Sheldrick, *Acta Crystallogr.*, 2008, **A64**, 112–122.

13 G. M. Sheldrick, *SHELXL-97, Program for Crystal Structure Refinement*, Universität Göttingen, 1997.

14 C. B. Hübschle, G. M. Sheldrick, B. Dittrich, *J. Appl. Crystallogr.*, 2011, **44**, 1281–1284.

15 K. Brandenburg, *Diamond 4.3.2*, Crystal Impact GbR, Bonn, Germany, 1997-2017.
